# Supplementary material for: Deciphering DED assembly mechanisms in FADD-procaspase-8-cFLIP complexes regulating apoptosis
Source: Nat Commun. 2024 May 6;15:3791. doi: 10.1038/s41467-024-47990-2 (PMC11074299; doi:10.1038/s41467-024-47990-2)
Supplement: Supplementary file 1 — Supplementary Information [file 41467_2024_47990_MOESM1_ESM.pdf]

Supplementary information

Deciphering DED Assembly Mechanisms in FADD-Procaspase-8-cFLIP Complexes Regulating Apoptosis

Chao-Yu Yang[1], Chia-I Lien[2], Yi-Chun Tseng[1,3,6], Yi-Fan Tu[3,6], Arkadiusz W. Kulczyk[4,6], Yen-Chen Lu[3], Yin-Ting Wang[1], Tsung-Wei Su[1], Li-Chung Hsu[2,5*], Yu-Chih Lo[3*], Su-Chang Lin[1,7*]

[1]Genomics Research Center, Academia Sinica, Taipei 11529, Taiwan

[2]Institute of Molecular Medicine, College of Medicine, National Taiwan University, Taipei 10002, Taiwan

[3]Department of Biotechnology and Bioindustry Sciences, College of Bioscience and Biotechnology, National Cheng Kung University, Tainan 70101, Taiwan

[4]Institute for Quantitative Biomedicine, Rutgers University, Department of Biochemistry and Microbiology, Rutgers University, Piscataway, NJ 08854, USA

[5]Graduate Institute of Immunology, College of Medicine, National Taiwan University, Taipei 10002, Taiwan

[6]Equal Contributions

[7]Lead Contact

*Correspondence:

Su-Chang Lin, tomlin@gate.sinica.edu.tw

Yu-Chih Lo, gracelo@ncku.edu.tw

Li-Chung Hsu, lichunghsu@ntu.edu.tw

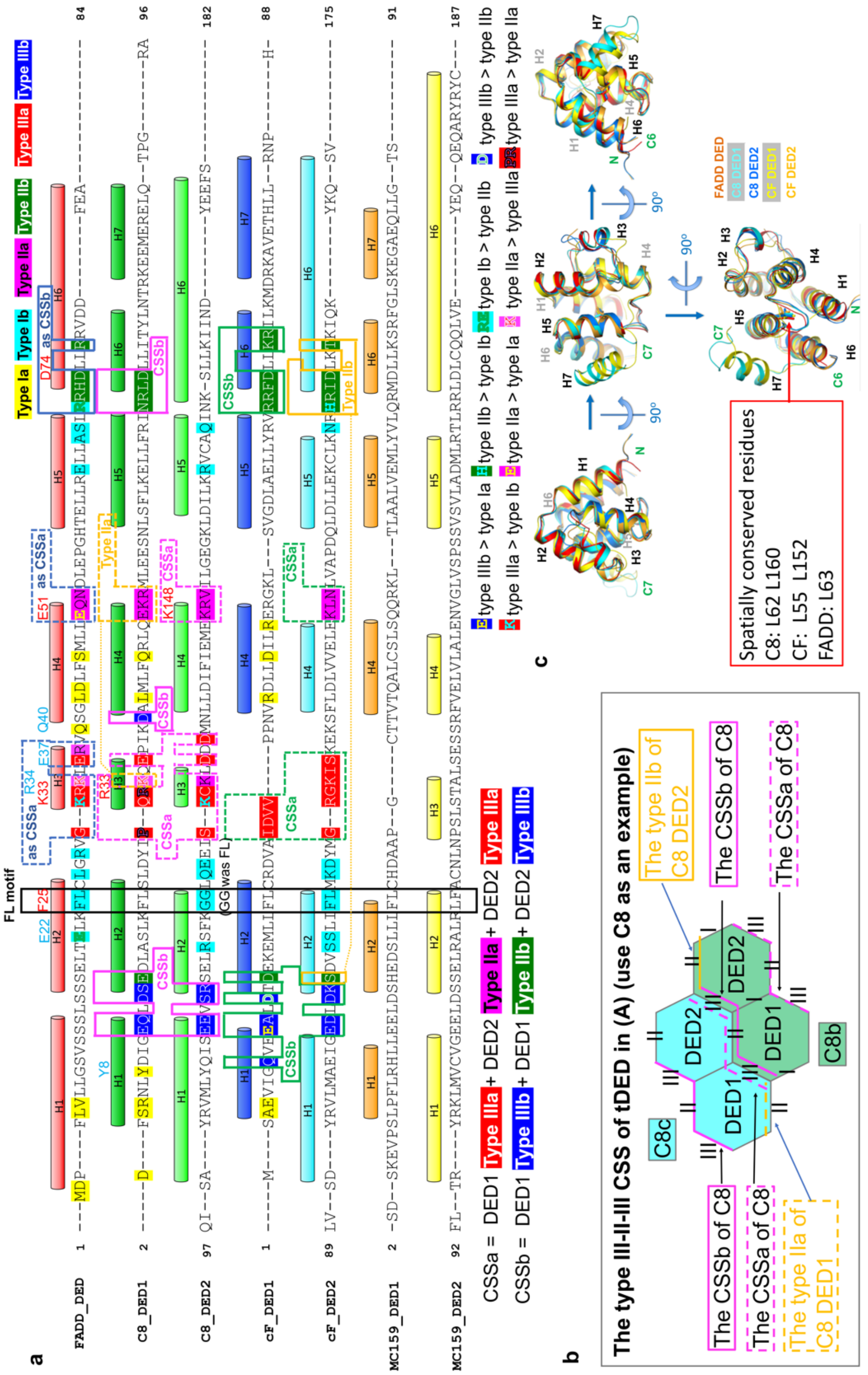

Supplementary Fig. 1: Structure-based sequence alignment and structure comparison of DED.

a, Structure-based alignment for the DED sequences of FADD, Casp-8 (C8), cFLIP (CF), and MC159. Representative interface residues are color-coded based on their locations on type Ia,

Ib, IIa, IIb, IIIa, or IIIb surfaces in the crystal structure. Residues are highlighted if they are involved in two surfaces, as shown in the lower right corner. Helices 1 to 7 of Casp-8^{tDED} are denoted as H1 to H7. The helices of cFLIP and MC159 are numbered to match the helices in Casp-8. The locations of FL motifs are indicated. Note that Casp-8 DED2 in this study has FL/GG mutations. Residues used for mutagenesis are shown above the sequence with red ones critical for signaling or assembly. The mutagenesis results for Casp-8 residues R33 and K148 are based on previous result[1]. The dashed boxes and solid boxes highlight the residues on the CSSa and CSSb, respectively, whereas the orange ones highlight the type II surfaces not involved in the type III-II-III CSS. In FADD^{DED}, the residues in dashed boxes and solid boxes assemble the surfaces mimicking CSSa and CSSb, which are labeled "as CSSa" and "as CSSb", respectively. See (**b**) for the locations of CSSa and CSSb.

b, Shows a representative tDED-tDED interaction via CSS between two Casp-8^{tDED} colored in cyan and green, respectively. Each hexagon represents a DED, with six sides representing the type I, II, and III surfaces. Angled/dashed pink lines and angled/solid pink lines highlight CSSa and CSSb of tDED, respectively, while the orange lines highlight the type II surfaces not involved in the type III-II-III CSS.

c, Structure comparison of the DEDs from FADD, cFLIP, and Casp-8. The cFLIP^{tDED} structure closely resembles Casp-8^{tDED}, except for a shorter loop H4-H5 in cFLIP DED1 and disordered H3 in both cFLIP DED1 and DED2 (Supplementary Fig. 9). The Cα atoms of spatially conserved Leu residues, shown as sticks, of all DEDs were used to generate balls in drawing different connectivity in Fig. 2 (see Methods). N, N terminus. C6 and C7, C-termini of H6 and H7, respectively.

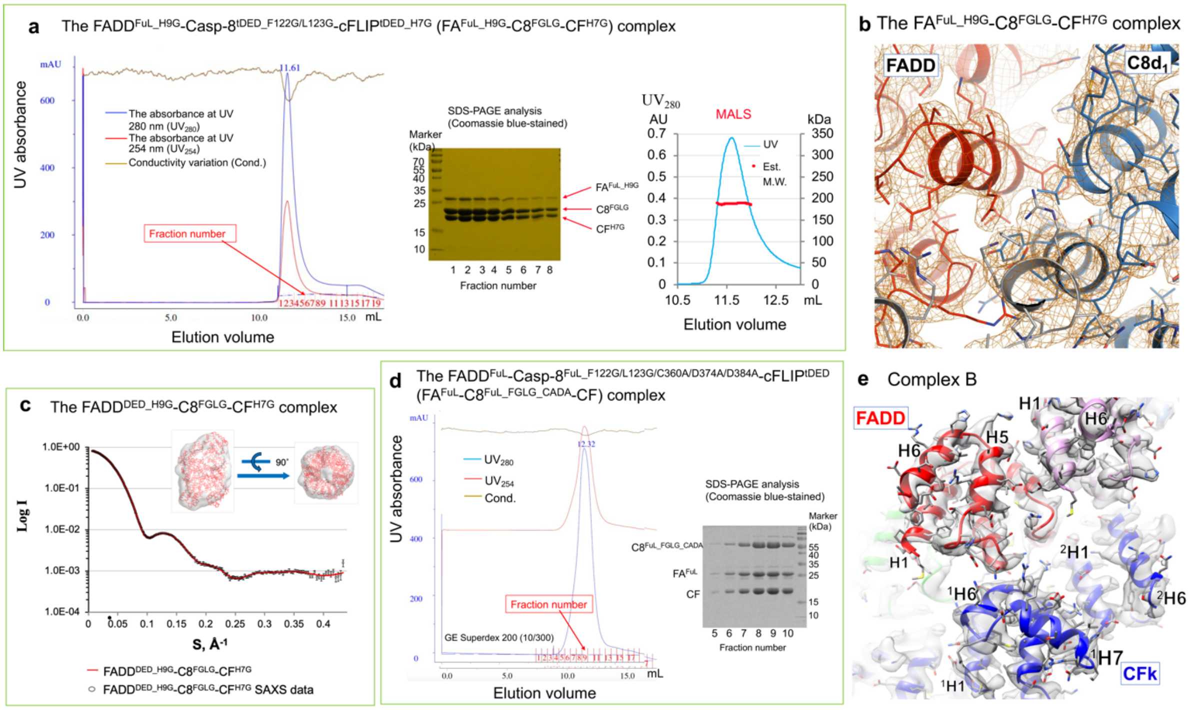

Supplementary Fig. 2: Characterizations of the ternary complexes.

a, Gel filtration profile of the FADD^{FuL_H9G}-Casp-8$^{tDED_F122G/L123G}$-cFLIP^{tDED_H7G} (FA^{FuL_H9G}-C8^{FGLG}-CF^{H7G}) complex in the SEC-MALS experiment done with a Superdex 200 increase (10/300 GL) column. The corresponding SDS-PAGE for the peak fractions and MALS result are shown. The MALS result shows that the apparent molecular mass is about 187.7 kDa, while the theoretical molecule weight of the 4:5:1 and 4:4:1 complex is about 217 and 195 kDa, respectively, suggesting that an end tDED molecule could have been lost at a low concentration in MALS. AU, absorbance units. Uncropped SDS-PAGEs are provided as a Source Data file.

b, Electron density map of the FA^{FuL_H9G}-C8^{FGLG}-CF^{H7G} complex, highlighting the type I interface between FADD and the C8d shown in Fig. 2a.

c, Fitting of SAXS data and theoretical curve derived from the corresponding FADD^{tDED_H9G}-C8^{FGLG}-CF^{H7G} complex structure. Please note that FADD in the sample for the SAXS experiment contains DED only. The fitting of the corresponding SAXS envelop and the crystal structure is also shown.

d, Gel filtration profile of the FADD^{FuL}-Casp-8$^{FuL_F122G/L123G/C360A/D374A/D384A}$-cFLIP^{tDED} complex (the FA^{FuL}-C8^{FuL_FGLG_CADA}-CF complex or complex A/B) and SDS-PAGE of peak fractions.

e, Cryo-EM envelop of the complex B, showing the 1st and 2nd FADD and CFk in the complex in Fig. 2b. The envelopes clearly show that FADD can be identified because there is no envelop for H7, while there is envelop for the cFLIP H7. Helices 1 to 7 of each DED are numbered H1 to H7, while ^{2}H4 stands for the helix 4 in the DED2.

Biochemical data were repeated at least twice with similar results. Uncropped blots are provided as a Source Data file.

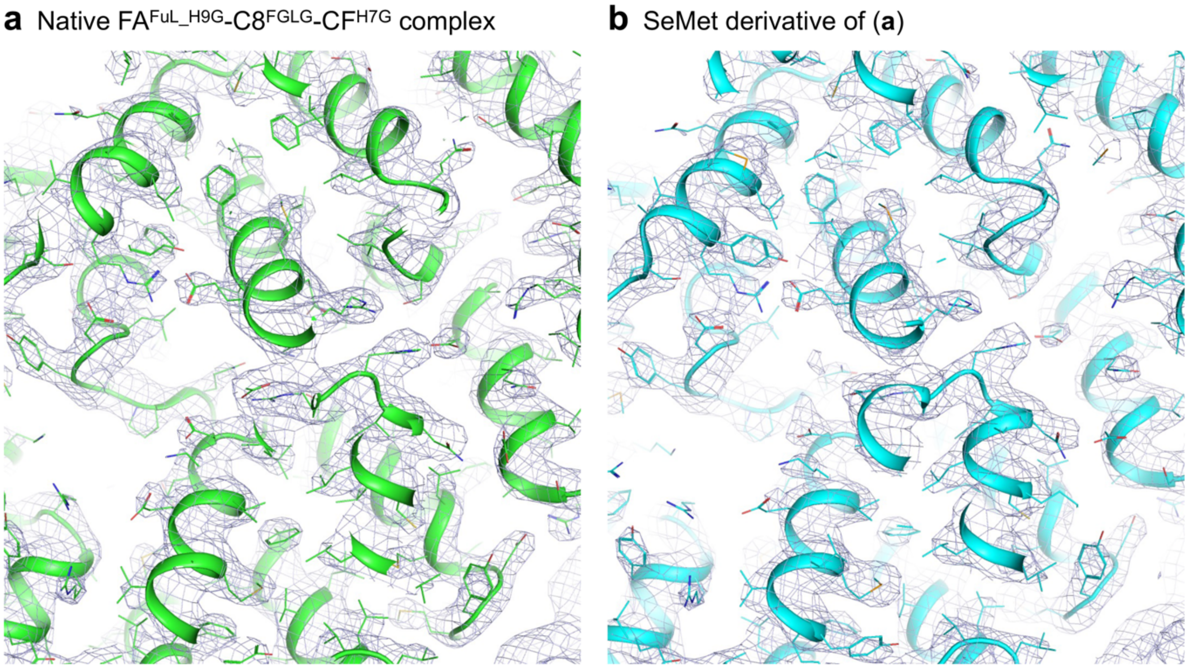

Supplementary Fig. 3: A portion of the electron density map with the crystal structure of the single-FADD ternary DED complex

a, The 2Fo-Fc map, contoured at 1.5 sigma by PyMol, for the native FA^{FuL_H9G}-$C8^{FGLG}$-CF^{H7G} complex.

b, The 2Fo-Fc map, contoured at 1.5 sigma by PyMol, for the SeMet derivative.

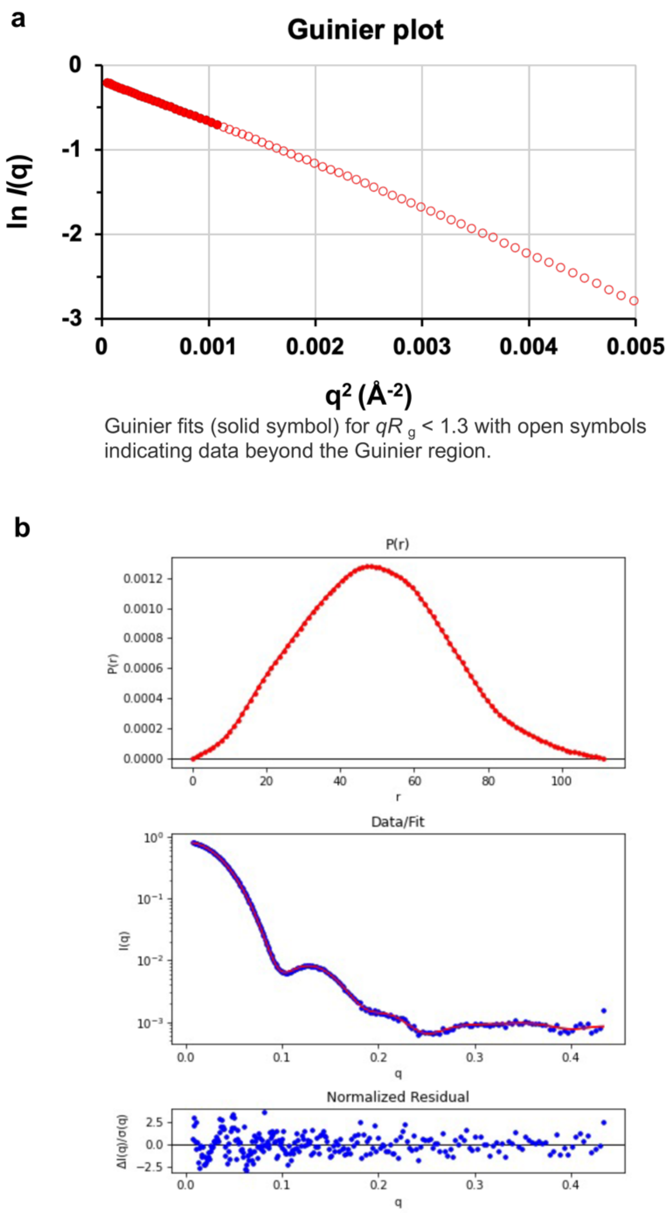

Supplementary Fig. 4: The SAXS data for the FADD^{tDED_H9G}-C8^{FGLG}-CF^{H7G} complex

a, The Guinier plot, see also Supplementary Fig. 2c.

b, *P*(*r*) analysis and fit of SAXS data to the model, see also Supplementary Table 2.

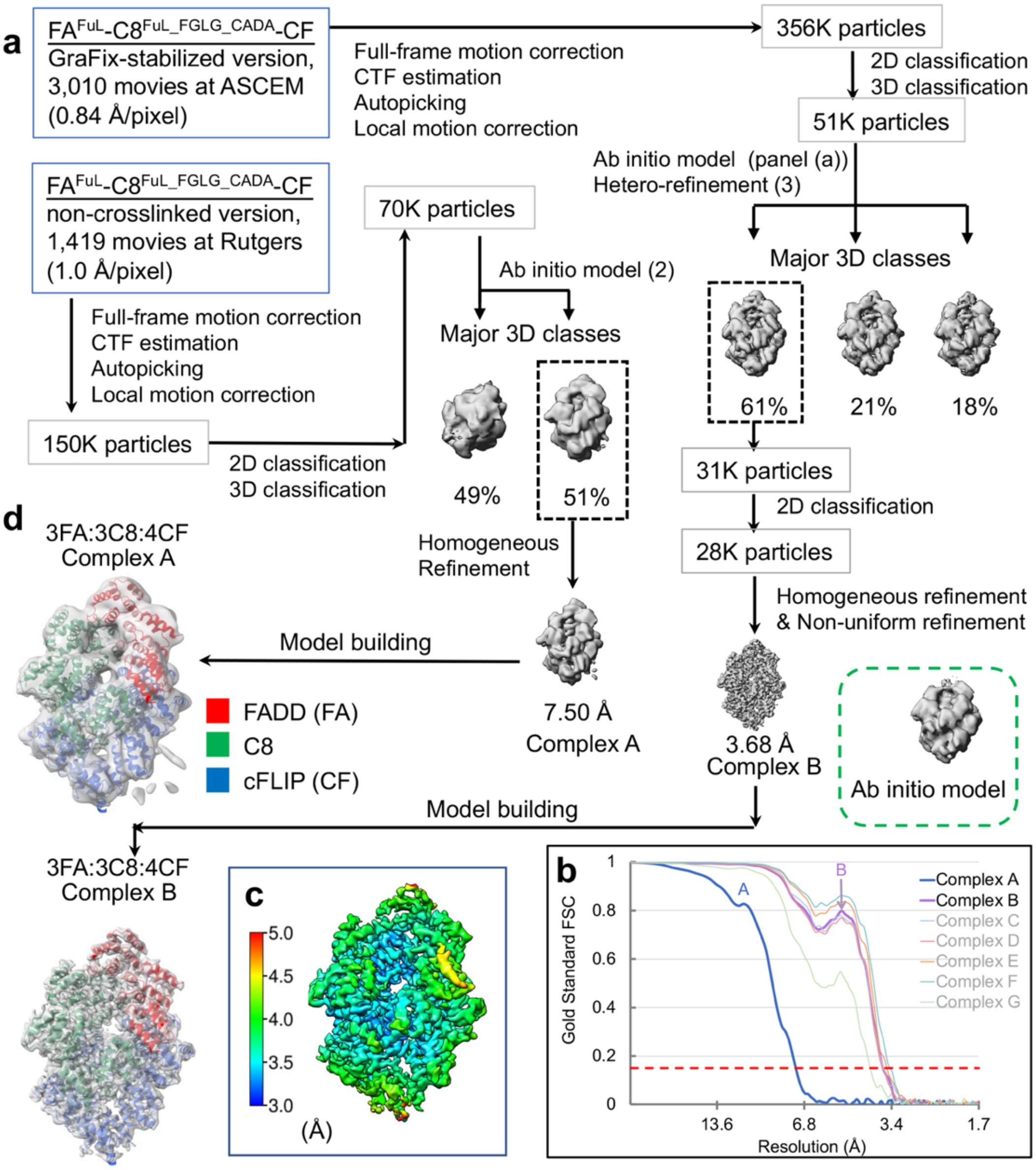

Supplementary Fig. 5: The procedure of cryo-EM structure determination

a, Diagrams showing the structure determination procedure of the FA^{FuL}-C8^{FuL_FGLG_CADA}-CF complex, leading to the envelops of Complexes A and B. Green dashed box shows Ab initio model.

b, Comparison of the FSC curves of all related cryo-EM DED structures solved by us.

c, Local resolution mapped onto the cryo-EM envelope of Complex B.

d, Overview of the models of the ternary FADD-Casp-8-cFLIP DED complexes fit into the corresponding cryo-EM density maps. See also Supplementary Fig. 7-9.

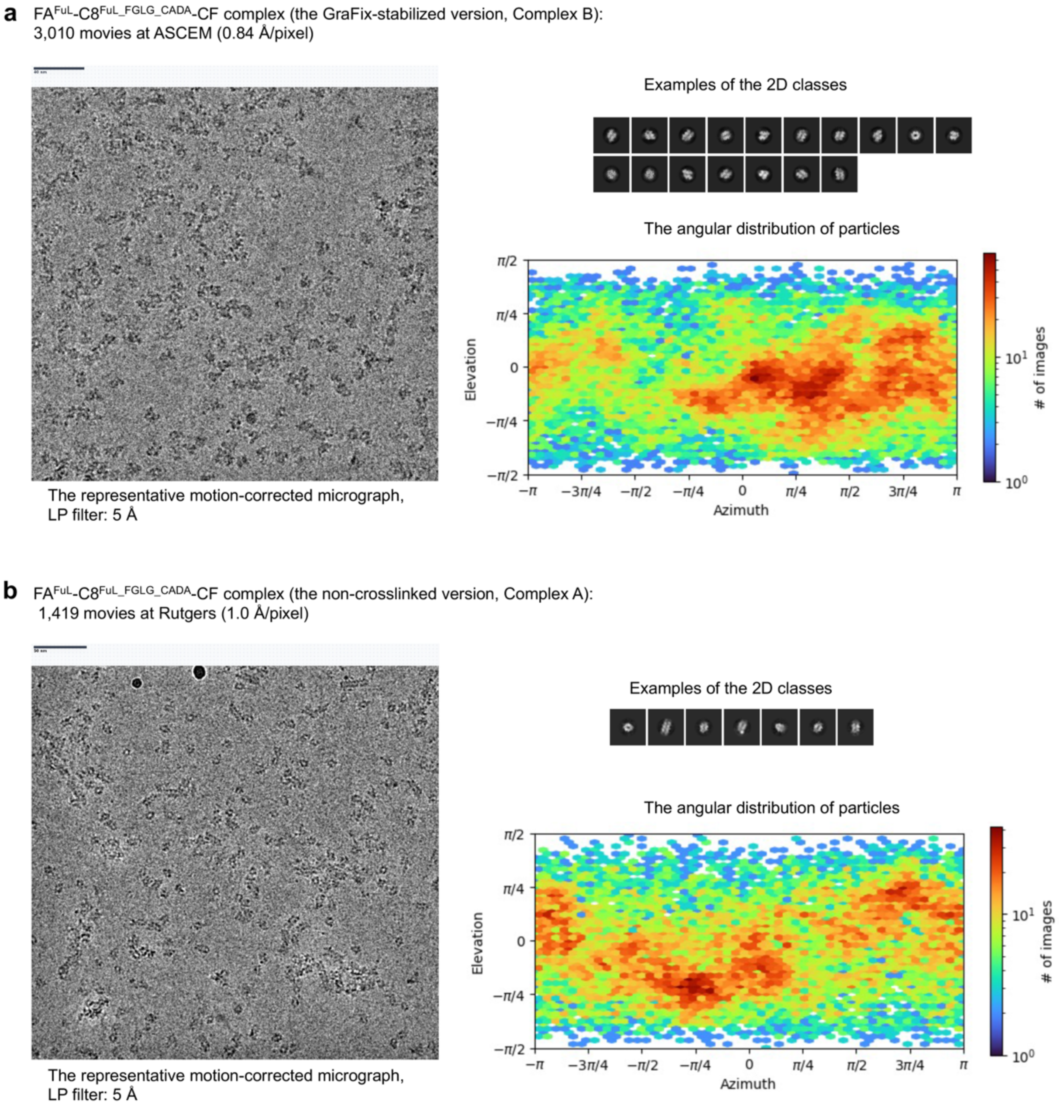

Supplementary Fig. 6: Representative Cryo-EM micrographs, examples of the 2D classes, and angular distribution of particles

a, Representative Cryo-EM micrographs, 2D classes, and angular distribution of particles in the cryo-EM analysis of the GraFix-stabilized version of the FA^{FuL}-$C8^{FuL\_FGLG\_CADA}$-CF complex (Complex B). See also Supplementary Fig. 5. Scale bar = 40 nm.

b, Same as (**a**), but the non-crosslinked version of the complex (Complex A). Scale bar = 50 nm. Thousands of movies were collected to produce micrographs with similar results. Source data are provided as a Source Data file.

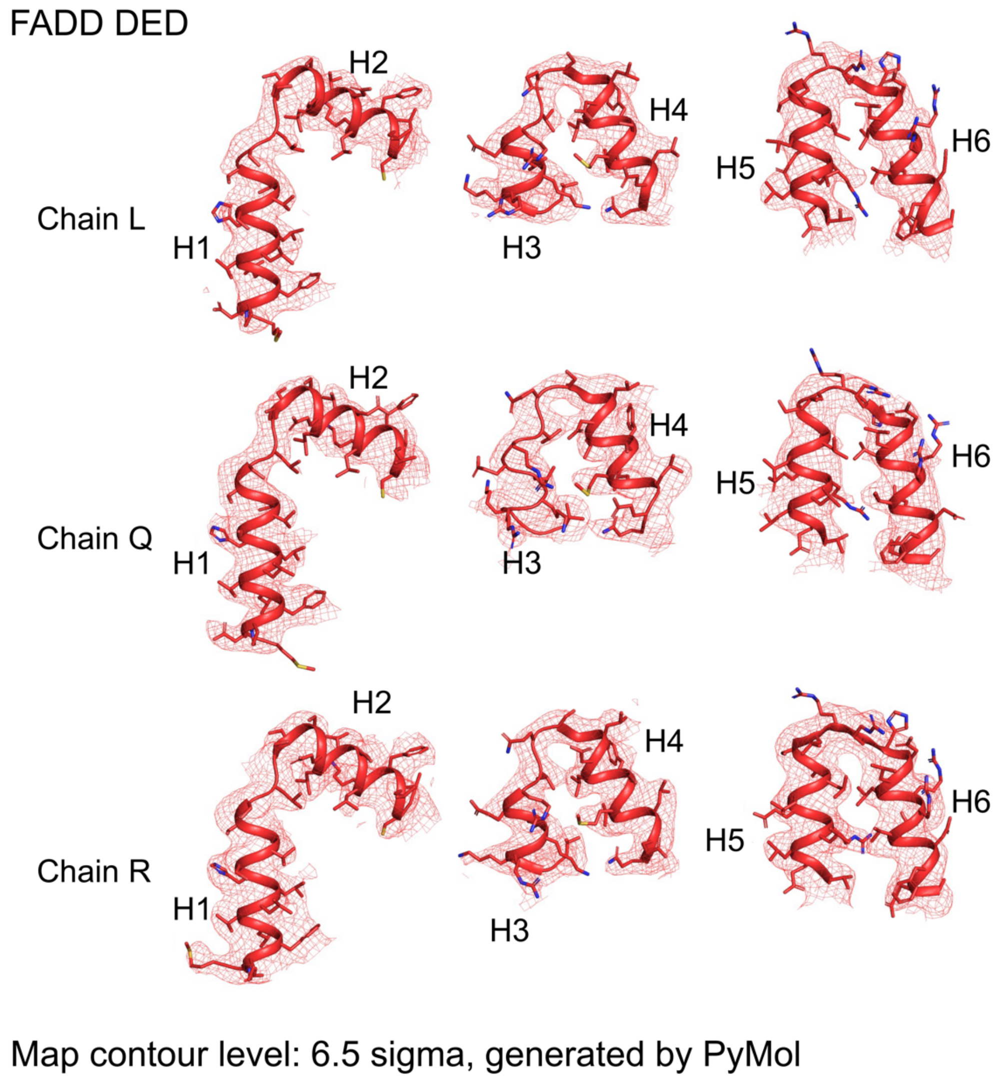

Supplementary Fig. 7: Cryo-EM maps for each helix of FADD DED

Shows cryo-EM maps for each helix of FADD DED in the model of the triple-FADD ternary DED complex

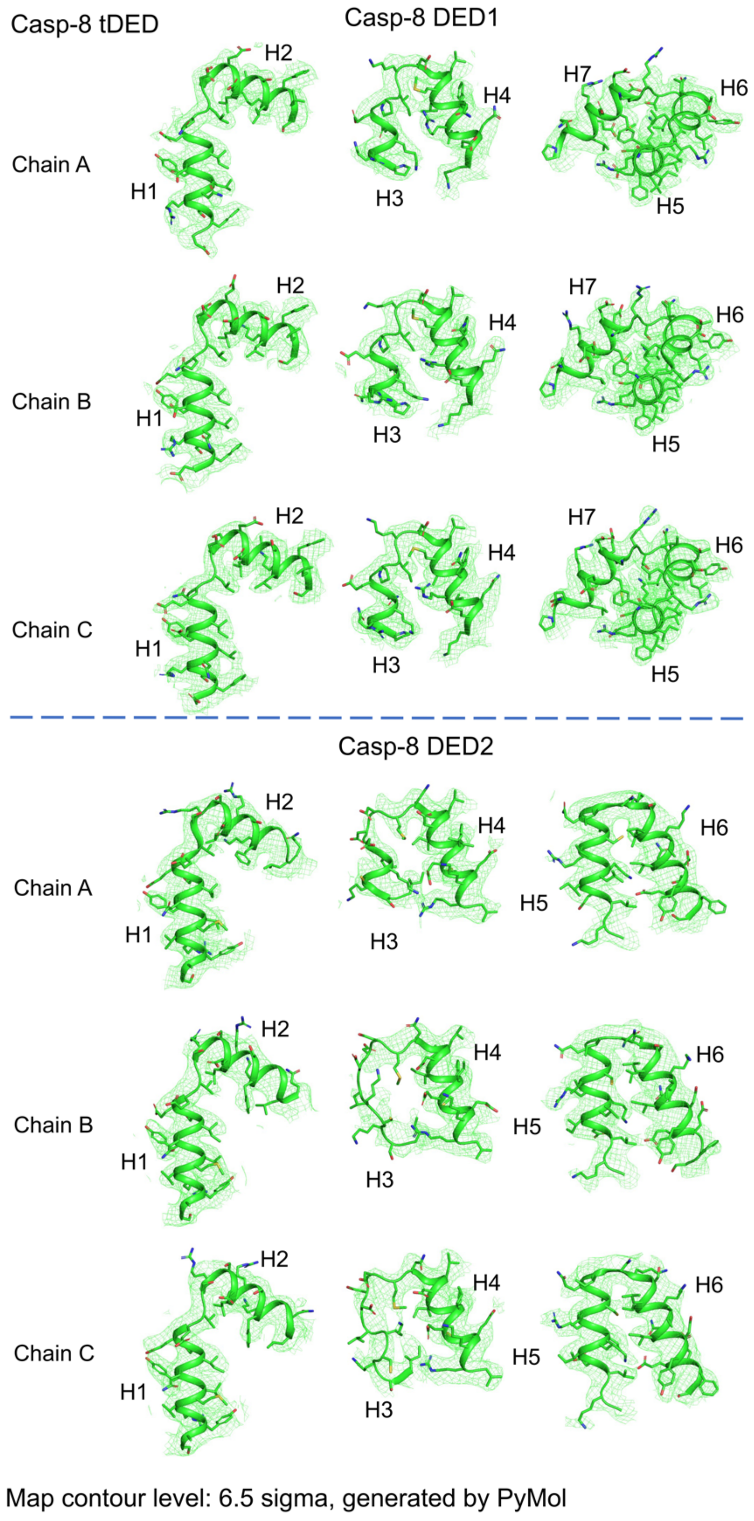

Supplementary Fig. 8: Cryo-EM maps for each helix of Casp-8 tDED

Shows cryo-EM maps for each helix of Casp-8 DED in the model of the triple-FADD ternary DED complex

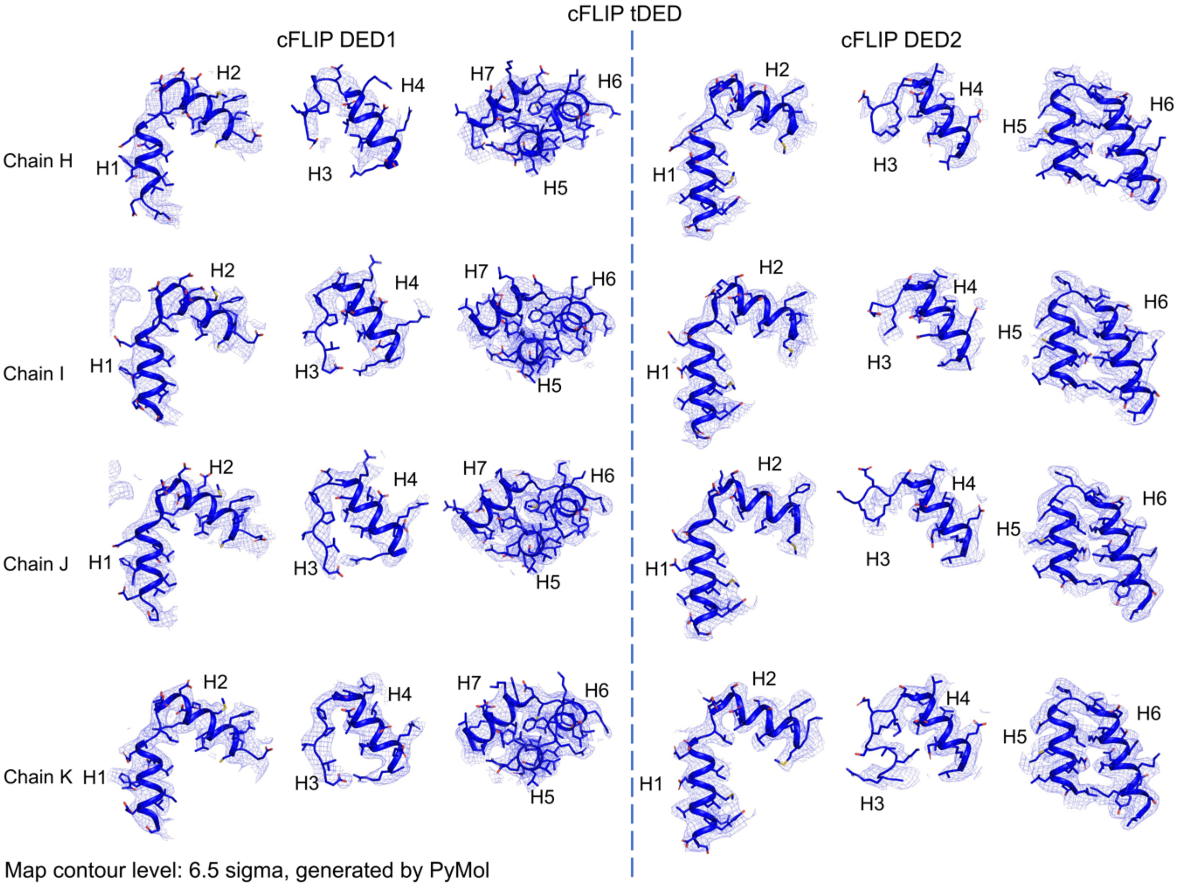

Supplementary Fig. 9: Cryo-EM maps for each helix of cFLIP tDED

Shows cryo-EM maps for each helix of cFLIP DED in the model of the triple-FADD ternary DED complex

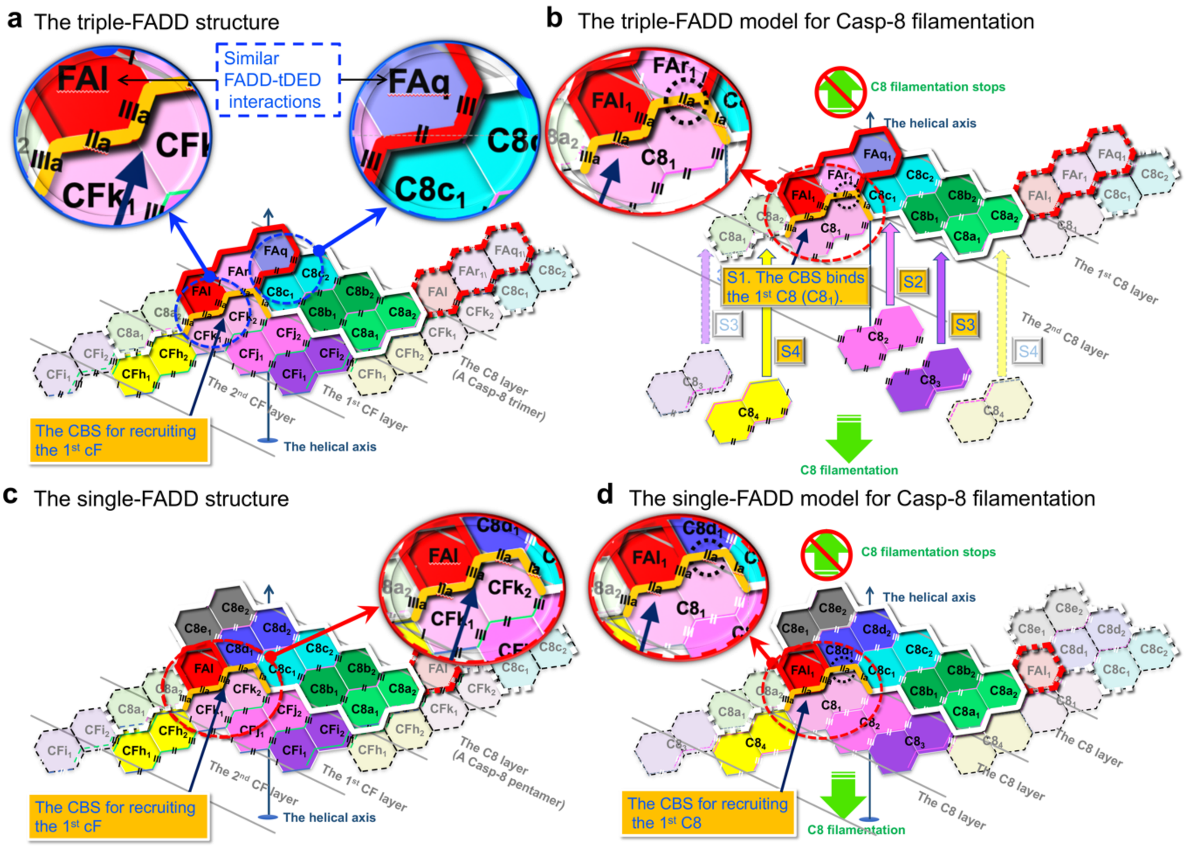

Supplementary Fig. 10: Two unidirectional FADD-mediated Casp-8 filamentation models for full Casp-8 activation

a, Blue ellipses highlight conserved type II and III interactions between the FADD-cFLIP and FADD-Casp-8 interfaces in the triple-FADD complex. Thick orange lines portray the CBS for recruiting the first cFLIP.

b, A model for triple-FADD-mediated Casp-8 filamentation, derived from the structure in (**a**) by substituting cFLIP^{tDED} with Casp-8^{tDED}. Casp-8^{tDED} molecule C8$_1$ targets the CBS (orange thick lines in red ellipses) of the triple-FADD-Casp-8 intermediate complex, marked as step S1, resulting in another CBS to bind the next Casp-8^{tDED} molecule C8$_2$ along type III connectivity, marked as step S2. Subsequently, the resultant CBS to bind Casp-8^{tDED} molecule C8$_3$ to complete the 2nd Casp-8 layer, marked as step S3. Steps S1-S3 are referred to as an

end-extending cycle. This 3-step process repeats, allowing three Casp-8 strands to sequentially extend their ends. For example, step S4 illustrates Casp-8^{tDED} molecule C8$_4$ targets the CBS of a Casp-8 double layer. Only the type II interaction between FADD^{DED} molecule FAr and Casp-8^{tDED} molecule C8$_1$, highlighted by a black dotted circle, has not been observed in any reported atomic coordinates.

c, Red ellipses highlight the conserved cFLIP-recruiting CBS observed in the single-FADD complex structure. Thick orange lines portray the CBS for recruiting the first cFLIP.

d, Similar to (**b**), but shows the model for single-FADD-mediated Casp-8 filamentation, derived from the structure in (**c**) by substituting cFLIP with Casp-8. Casp-8 filaments extend when Casp-8 is repeatedly added to the bottom end. Casp-8-binding CBS is shown as orange thick lines in red ellipses. Only the type II interaction between Casp-8^{tDED} molecules C8d and C8$_1$, highlighted by a black dotted circle, has not been observed in any reported atomic coordinates.

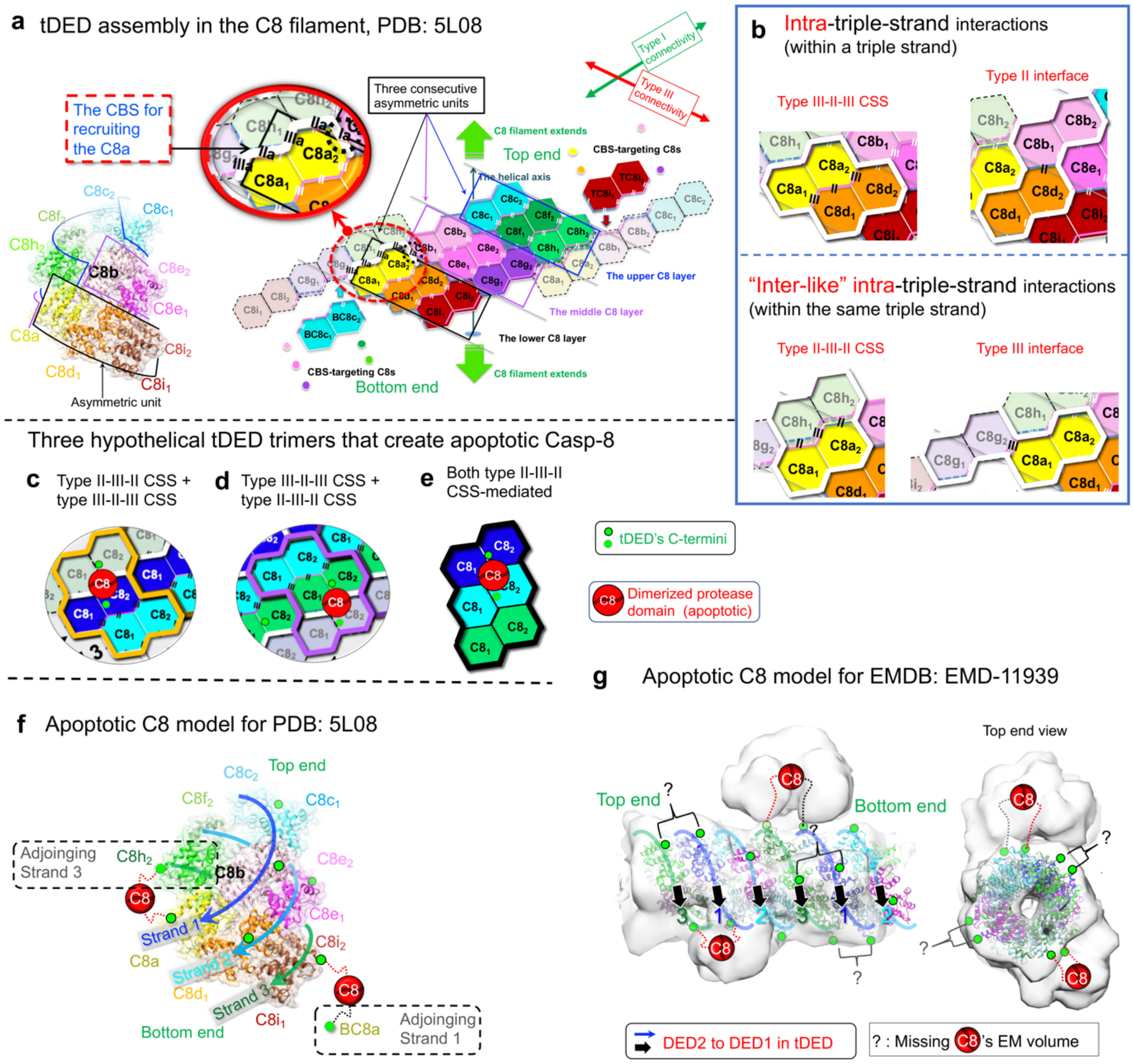

Supplementary Fig. 11: Possible Casp-8^{tDED} assemblies in different structures

a, Shows three consecutive asymmetric units to depict tDED assembly in the cryo-EM structure of Casp-8^{tDED} filaments (5L08[1] [http://doi.org/10.2210/pdb5L08/pdb]). Each asymmetric unit comprises a type III-II-III CSS-mediated non-apoptotic Casp-8 trimer, which would correspond to an offset trimer[2]. White thick lines in red ellipses portray the CBS, ~1,437 Å^2, of a Casp-8 double layer, which could bind Casp-8^{tDED} molecule C8a, similar to step S4 in Supplementary Fig. 10b. Subsequently, the resultant CBS sequentially recruits Casp-8^{tDED} molecules C8d, C8i, and BC8c along the type III connectivity to extend the bottom end. See

also (**f**) and Fig. 7c. The top end could extend in a similar way by recruiting Casp-8^{tDED} molecules, such as TC8i. For comparison, a black dotted circle highlights type I interface between C8b and C8a, which is only ~500 Å^2.

b, The intra-triple-strand and inter-like intra-triple-strand interactions occur in (**a**).

c-e, Three additional tDED trimers generated if Casp-8^{tDED} dimerizes randomly via either type II-III-II CSS or type III-II-III CSS. Apoptotic Casp-8 protease dimers are shown as red balls and labeled as C8.

f, Portrays Strands 1-3 of (**a**) along the type I connectivity, which exhibit the same helical twist of about 70° and the same intra-triple-strand contacts in (**b**). The first apoptotic Casp-8 protease dimer is formed between Casp-8^{tDED} molecules C8h and C8a, through inter-like intra-triple-strand contacts in (**b**). The second protease dimer is behind the model and, therefore, is not drawn, while the third one is form between Casp-8^{tDED} molecules C8i and BC8a.

g, Shows an oligomeric Casp-8 tDED model placed in the EM volume of the central region of the FADD-Casp-8 complex (EMD-11939[2] [https://www.ebi.ac.uk/pdbe/entry/emdb/EMD-11939]). Suggested DED2 to DED1 orientations are indicated by black arrows with suggested Strand IDs/numbers 1~3, based on the assumption that FADD binds the left end of the EM volume[2]. Notably, some aligned Casp-8^{tDED} dimers didn't generate the EM volume of a protease dimer, which are labeled by question marks, for an unknown reason. Further cryo-EM investigations are required to conclusively elucidate how tDED brings two Casp-8's protease domains together. See also Fig. 7c.

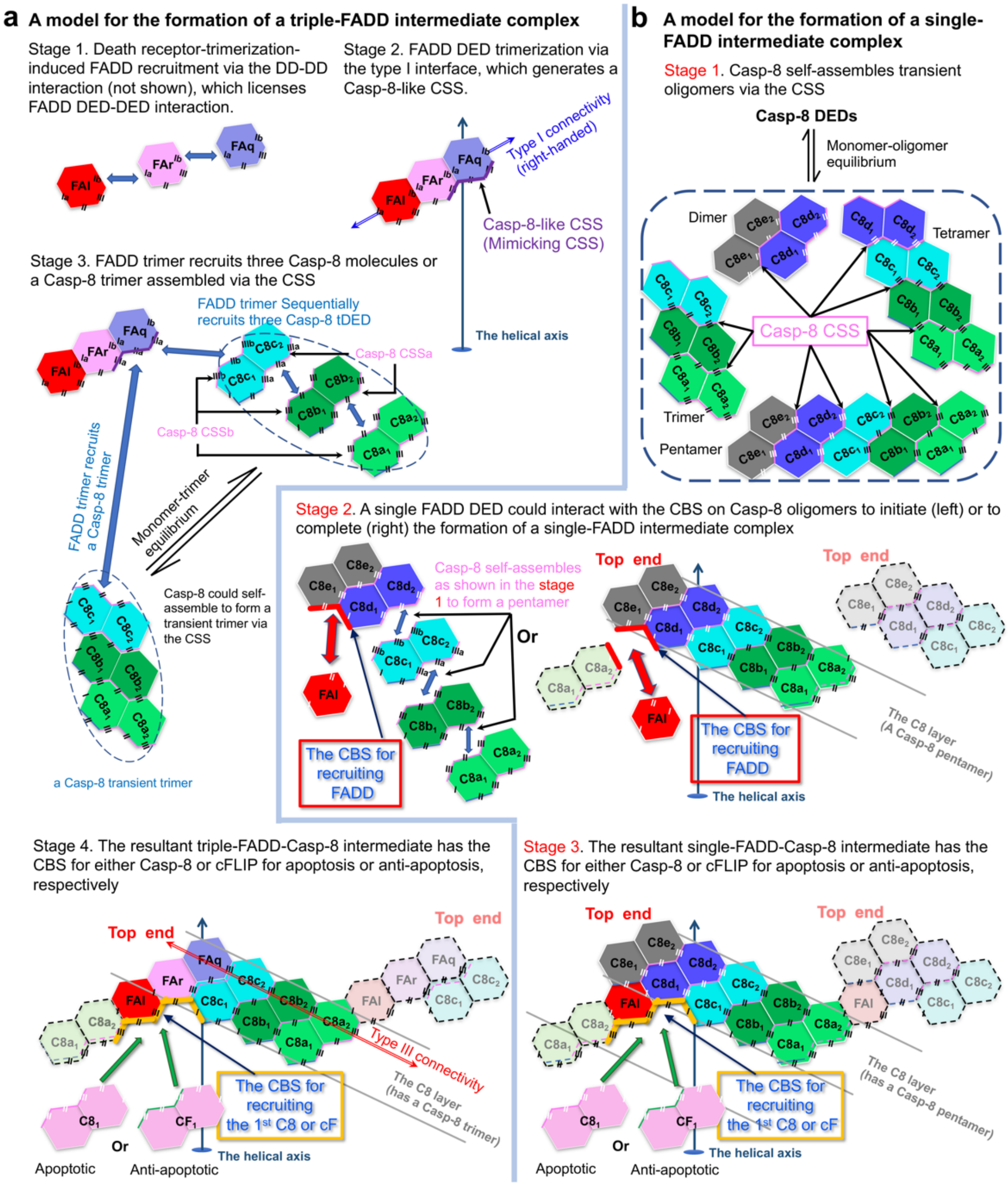

Supplementary Fig. 12: Two models for unidirectional FADD-mediated intermediate complex formation

a, This model depicts the formation of a triple-FADD intermediate complex. In stage 1, upon, for example, DR activation, FADD molecules are recruited to the receptor complex, and three FADD^{DED} interact via the type I interface. In stage 2, a right-handed FADD^{DED} trimer transiently forms, where FAr and FAq together create a Casp-8-like CSS for interacting with the Casp-8 CSS. Therefore, in stage 3, to form a FADD-Casp-8 intermediate complex, the

FADD trimer could sequential bind three Casp-8 molecules or a Casp-8 trimer. Either way, Casp-8^{tDED} assemble via the Casp-8 CSS, suggesting a potential monomer-oligomer equilibrium for Casp-8^{tDED}. In stage 4, the resultant triple-FADD-Casp-8 intermediate complex generates a CBS to recruit Casp-8 or to become a target for cFLIP. The hexagon diagrams, including hexagons, dashed hexagons, and angled and angled/dashed lines, were colored, labeled, and explained in Fig. 2a. See Supplementary Fig. 13 for the determination of the top and bottom ends of an oligomeric DED complex.

b, This model illustrates the formation of a single-FADD intermediate complex. As discussed in (**a**), Casp-8^{tDED} may exhibit monomer-oligomer equilibrium and could self-assemble via the Casp-8 CSS to generate different Casp-8 oligomers in stage 1. In stage 2, upon, for example, receptor activation, an exposed or activated single $FADD^{DED}$ interacts with and stabilizes a transient Casp-8^{tDED} pentamer to form an intermediate complex. Alternatively, a single $FADD^{DED}$ could also interact with a Casp-8^{tDED} dimer and then recruit three Casp-8^{tDED} via the Casp-8 CSS to form the same intermediate complex. In stage 3, the resultant single-FADD-Casp-8 intermediate complex generates a CBS to recruit Casp-8 or to become a target for cFLIP. The CBS generated by the single-FADD complex is quite similar to that in the triple-FADD complex shown in (**a**), except that two type IIa surfaces on the former are provided by FADD, whereas one on the latter is provided by Casp-8.

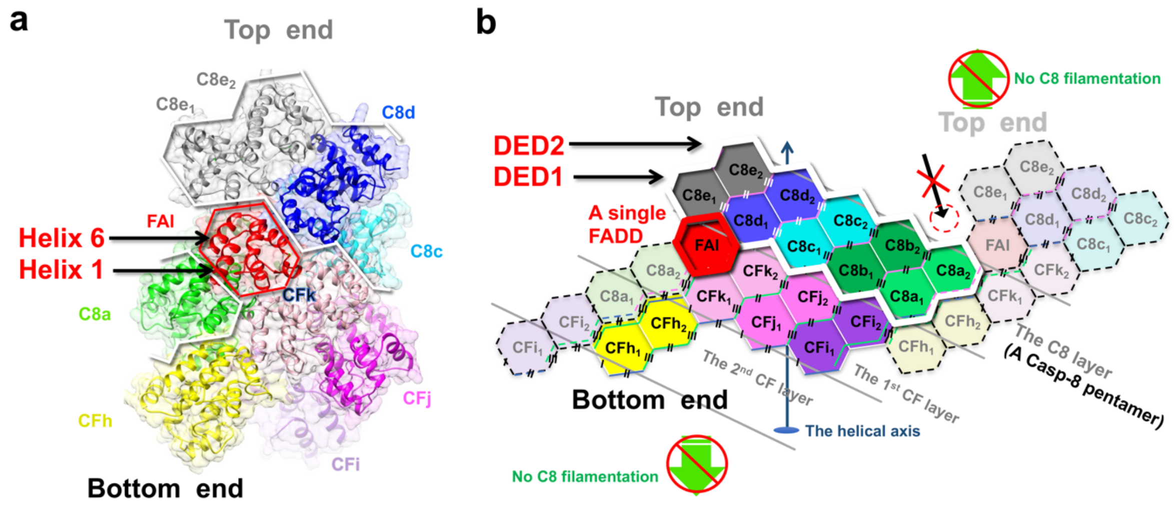

The FA^{FuL_H9G}-C8^{FGLG}-CF^{H7G} 1:5:4 complex

Supplementary Fig. 13: The top and bottom ends of a multiprotein oligomeric DD-fold complex

a, The designation of the top end and bottom end of a DD-fold oligomeric core complex is established based on the positioning of helix 1 (or N-terminus) and helix 6 (or C-terminus) in each DD-fold molecule of the oligomeric complex. This determination is made considering that helix 1 consistently resides closer to the bottom end than helix 6. As shown here using our single-FADD ternary complex as an example, the top end molecule is Casp-8^{tDED} molecule C8e, while the bottom end molecule is cFLIP^{tDED} molecule CFh.

b, Likewise, within the tDED complex, DED1 of each tDED consistently aligns closer to the bottom end than DED2. This consistent N to C orientation observed in the oligomeric DD-fold core complex structure remains a shared characteristic across various cellular complexes, including the Myddosome, PIDDosome, RIG-I-MAVS signalosome, and apoptosome.

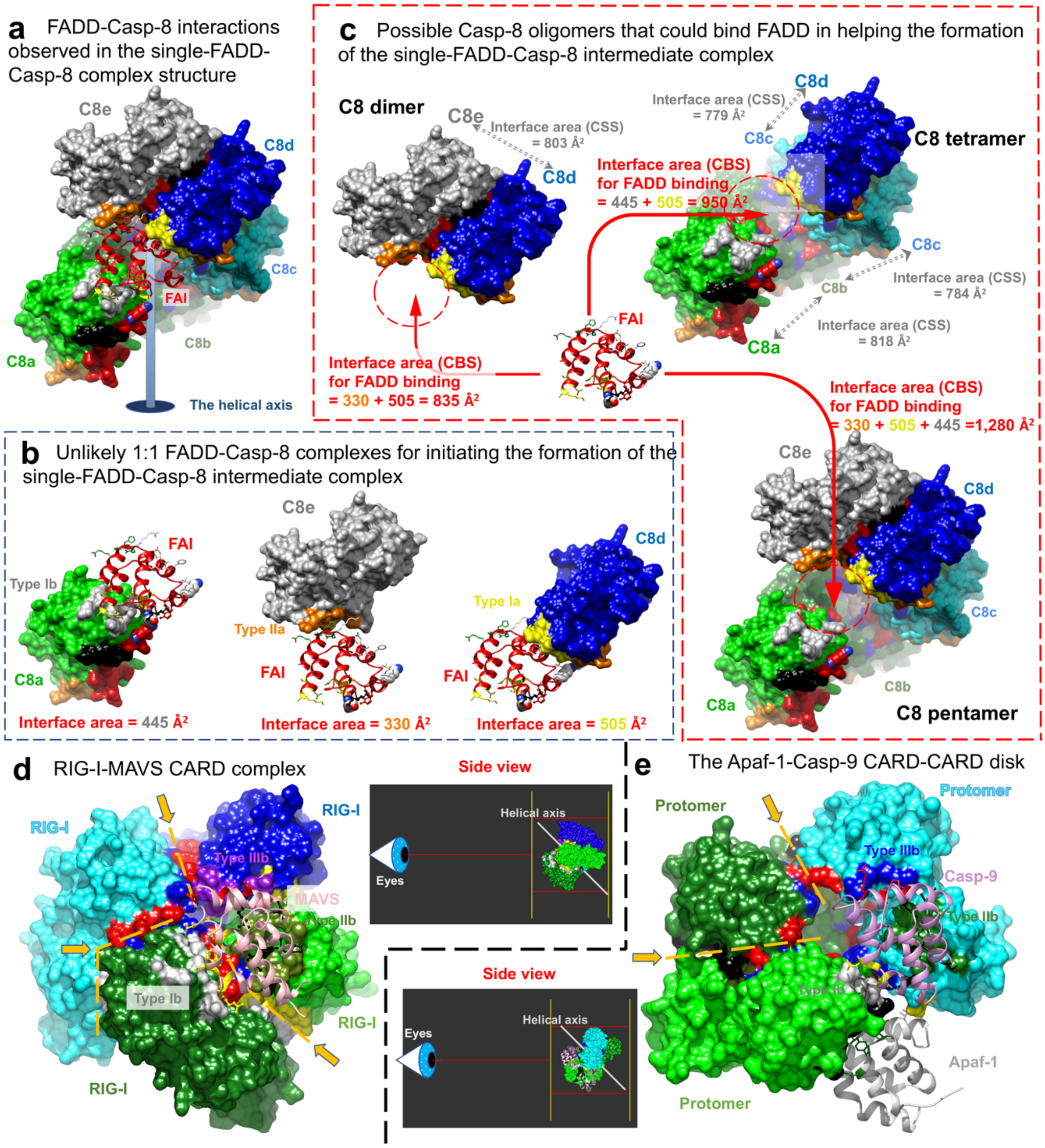

Supplementary Fig. 14: Potential FADD-Casp-8 interactions in initiating single-FADD Casp-8 intermediate complex formation

a, Depicts FADD-Casp-8 interactions observed in the structure of the single-FADD-Casp-8 complex. FADD and Casp-8 are labeled and colored as in Fig. 2a.

b, Illustrates three conceivable 1:1 FADD-Casp-8 transient complexes derived from (**a**). These are less stable and, consequently, less likely to assemble compared to those shown in (**c**). The

FADD-Casp-8 interface size is indicated and also color-coded to corresponding surface on Casp-8.

c, Displays three potential Casp-8 transient oligomers self-assembled via its CSS. The FADD-Casp-8 interface areas in these structures are significantly larger than those shown in (**b**). Consequently, self-assembled Casp-8 oligomers could generate a CBS to bind a single FADD, thereby stabilizing and forming the single-FADD-Casp-8 intermediate complex.

d, Illustrates that RIG-I^{tCARD} utilizes the type III-I-III CSS to form a tetramer. Orange arrows and broken lines indicate the approximate locations of the type III-II-III CSS. The resultant composite single-CARD binding site that binds a single MAVS CARD, shown in ribbons, comprises a type Ib, a type IIb, and a type IIIb surfaces. The binding of a single MAVS CARD, in turn, generates another CBS to bind an additional MAVS CARD, triggering a chain reaction for MAVS filament assembly. The type I, II, and III surfaces are colored as those on Casp-8, except that the type IIIb surface of the blue Casp-8 is colored in purple. The side view depicts the helical axis (white) and sightline (red).

e, Shows that the type III-II-III CSS of the Apaf-1^{CARD}-Casp-9^{CARD} protomer brings three Apaf-1-Casp-9 CARD protomers together to create composite single-CARD binding sites. During the assembly of the CARD-CARD disk of the human apoptosome, this site that binds a single Casp-9 CARD, shown in pink ribbons, would include a type Ib, a type IIb, and a type IIIb surfaces. Orange arrows and broken lines indicate the approximate locations of the type III-II-III CSS. The type I, II, and III surfaces are colored as those on Casp-8. The side view displays the helical axis (white) and sightline (red).

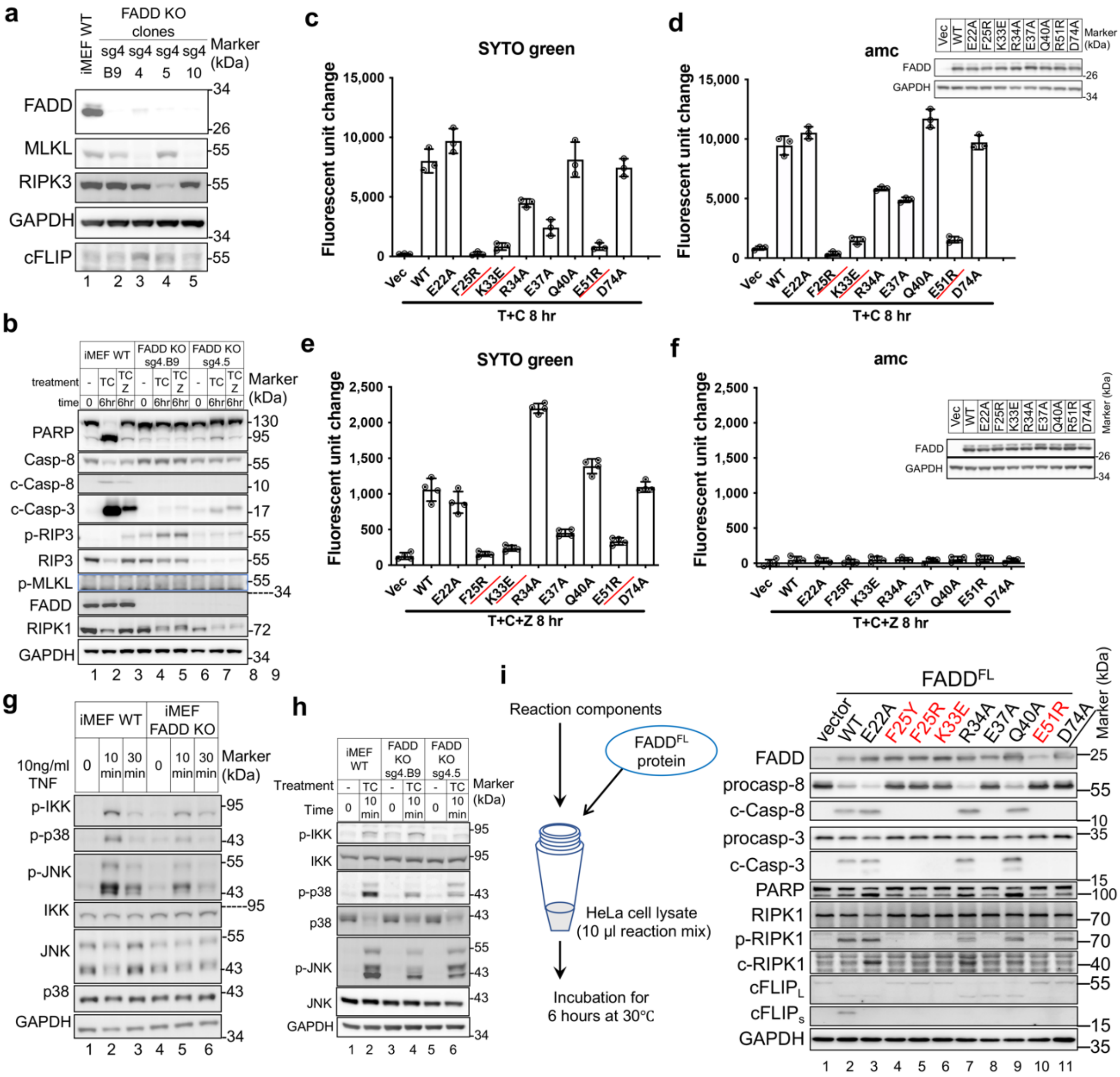

Supplementary Fig. 15: Roles of the triple- and single-FADD models in hierarchical apoptotic signaling

a, Western blotting analysis of protein expression levels of FADD and selected signaling proteins involved in TNFR signaling across various clones of FADD-deficient immortalized mouse embryonic fibroblasts (iMEF). The results are compared to those obtained from wild-type iMEF, labeled as iMEF WT or WT iMEF, cells.

b, Examination of TNF-induced apoptotic and necroptotic signals in WT iMEF and *FADD*$^{-/-}$ iMEF clone sg4.B9 through western blotting. Cells were pre-treated with CHX (10 μg/ml) for

30 minutes, followed by TNF (10 ng/ml) treatment with or without z-VAD-fmk (50 ng/ml). T, TNF; C, Cycloheximide; Z, z-VAD-fmk.

c, Fluorescence apoptosis/necrosis assays for cells treated with TC from Fig. 6b at 8 hours, measuring cell death presented by SYTO green signals.

d, Fluorescence apoptosis/necrosis assays for TC-treated cells in **c**, measuring the activated caspase-3 level presented by amc signals. FADD mutant protein expression levels were examined by western blotting. Error bars in (**c**) and (**d**) represent standard deviation, n = 3 independent replicates.

e, Same as (**c**), except that the cells (treated by TCZ) are from Fig. 6c at 8 hours.

f, Same as (**d**), except that the cells (treated by TCZ) are from Fig. 6c at 8 hours. Error bars in (**e**) and (**f**) represent standard deviation, n = 4 independent replicates.

g, Western blotting analysis of TNF (10 ng/ml)-induced NF-κB and MAPK signals in WT iMEF and $FADD^{-/-}$ iMEF clone sg4.B9.

h, Similar to (**g**), except that the cells were pre-treated with CHX (10 μg/ml) for 30 minutes before TNF treatment (10 ng/ml) for 6 hours.

i, Addition of FADD simultaneously induces two functionally distinct complexes for Casp-8 activation and RIPK1 phosphorylation in HeLa cell lysate-based mutagenesis assays, examined by western blotting.

All the Western blotting data were repeated twice with similar results. Uncropped blots are provided as a Source Data file.

Supplementary Table 1: X-ray data collection, phasing and refinement statistics for the single-FADD ternary DED complex

	FA^{FuL_H9G}-$C8^{FGLG}$-CF^{H7G}*	FA^{FuL_H9G}-$C8^{FGLG}$-CF^{H7G} (Se)
**Data collection**		
Space group	P21 21 21	P21 21 21
Cell dimensions		
a, *b*, *c* (Å)	113.170, 149.680, 175.683	113.963, 150.057, 175.703
α, β, γ (°)	90.00, 90.00, 90.00	90.00, 90.00, 90.00
		Peak
Wavelength	0.99984	0.97939
Resolution (Å)	30.09-3.10 (3.21-3.10)**	30.00-3.32 (3.44-3.32)
R_{sym} or R_{merge}	0.078 (0.917)	0.124 (0.554)
$I / \sigma I$	21.645 (1.750)	20.228 (3.298)
Completeness (%)	99.7 (97.5)	99.5 (100)
Redundancy	6.1 (5.6)	9.6 (5.5)
**Refinement**		
Resolution (Å)	30.09-3.11 (3.22-3.11)	29.81-3.32 (3.44-3.32)
No. reflections	53,677 (4,983)	44,772 (4,378)
R_{work} / R_{free}	0.1995/0.2413	0.1939/0.2311
No. atoms		
Protein	13,754	13,822
Ligand/ion	-	1
Water	-	-
B-factors		
Protein	116.0	112.4
Ligand/ion	-	147
Water	-	-
R.m.s deviations		
Bond lengths (Å)	0.002	0.002
Bond angles (°)	0.435	0.428
PDB ID	8YD8	8YD7

*One crystal used for each data set.
**Values in parentheses are for highest-resolution shell.

Supplementary Table 2: SAXS data collection and structural parameters of $FADD^{DED\_H9G}$-$C8^{FGLG}$-CF^{H7G} complex

Sample details	
Organism	Human
source	*E. coli* expressed
UniProt sequence ID (residues in construct)	Q13158(1-84); Q14790(1-185); O15519(1-181)
Extinction coefficient [A 280, 0.1%(w/v)]	0.344
Protein partial specific volume (cm^3 g^{-1})	0.7657
Particle contrast, $\Delta\rho$ [$\rho_{protein} - \rho_{solvent}$; 10^{10} cm^{-2}]	2.3976 [11.8704 - 9.4728]
M_r from chemical composition (Da)	203,130
C (mol/cm^3)	0.2075
Buffer solution	20 mM Tris-HC1 pH 8.0, 80 mM NaCl
SAS data collection parameters	
Instrument	Synchrotron 23A SWAXS endstation of NSRRC
Beam geometry	0.5 mm diameter beam
Wavelength (Å)	0.82825
q-range ($Å^{-1}$)	0.007-0.4336
Exposure time	300s with 30s single exposure time for 10 successive exposures
Concentration range (mg ml^{-1})	7.0
Temperature (K)	288
Data processing[3]	
Primary data reduction	NSRRC 23A SWAXS package
Data processing	*ATSAS* 2.8.1; *RAW* 2.2.2
Ab initio analysis	*RAW* 2.2.2, *ATSAS 3.2.1 (r14885)*
Extinction coefficient estimate	*ProtParam*
Validation and averaging	n/a
Rigid-body modelling	n/a
Computation of model intensities	*RAW* 2.2.2
Model χ^2	*9.0*
Three-dimensional graphics representation	*PyMOL*
Structural parameters	
Guinier analysis	
$I(0)$ (cm^{-1})	0.8271 ± 0.000506
R_g (Å)	37.8254 ± 0.0377
q_{min} ($Å^{-1}$)	0.0069
qR_g max (q_{min} = 0.0069 $Å^{-1}$)	1.2755
Coefficient of correlation, R^2	0.9998
M_r from *I(0)* (*ratio to calculated M*)	211,000 (1.04)
$P(r)$ analysis	
$I(0)$ (cm^{-1})	0.8264 ± 0.000140
R_g (Å)	37.4407 ± 0.00942
D_{max} (Å)	111.2924 ± 1.1798
q range ($Å^{-1}$)	0.0069-0.4336
χ^2 fit *(estimate from RAW)*	2.1002

M_r from *I(0) (ratio to calculated M)*	211,000 (1.04)
Porod Volume (Å^3) (*V_P/calculated M)*	237,000 (1.17)

Supplementary Table 3: Cryo-EM data collection, refinement and validation statistics for the triple-FADD ternary DED complex

	Complex A (EMD-39127)	Complex B (EMD-39126) (PDB 8YBX)
**Data collection and processing**		
Magnification	130K	165K
Voltage (kV)	200	300
Electron exposure (e$^-$/Å^2)	~50	~72
Defocus range (μm)	-0.5~-3.5	-0.5 ~ -2.5
Pixel size (Å)	1.00	0.84
Symmetry imposed	C1	C1
Initial particle images (no.)	150,000	356,000
Final particle images (no.)	21,535	28,685
Map resolution (Å)	7.56	3.68
FSC threshold	0.143	0.143
Map resolution range (Å)	5.1~10.3	2.2~6.4
**Refinement**		
Initial model used (PDB code)		
Model resolution (Å)		3.68
FSC threshold		0.143
Map sharpening *B* factor (Å^2)		-50
Model composition		
Non-hydrogen atoms		12,126
Protein residues		1482
Ligands		-
B factors (Å^2)		
Protein		101.68
Ligand		-
R.m.s. deviations		
Bond lengths (Å)		0.002
Bond angles (°)		0.400
Validation		
MolProbity score		1.29
Clashscore		5.14
Poor rotamers (%)		0
Ramachandran plot		
Favored (%)		97.94
Allowed (%)		2.06
Disallowed (%)		0

Supplementary Table 4: List of oligonucleotides used in this study.

Cloning primers	Seq (5'-3')	Purpose
hFADD_F_AS4	CCGCGATCGCGCTAGCATGGACCCGTTCCTG	pAS4.1w.Pbsd-aOn
hFADD_R_AS4	TATCTGTACAGCTAGCTCAGGACGCTTCGGAG	pAS4.1w.Pbsd-aOn
mFADD_F_AS4	CCGCGATCGCGCTAGCATGGACCCATTCCTGG	pAS4.1w.Pbsd-aOn
mFADD_R_AS4	TATCTGTACAGCTAGCTCAGGGTGTTTCTGAGGAAGAC ACAG	pAS4.1w.Pbsd-aOn
human FADD forward	GATCCCATGGACCCGTTCCTGGTGCTG	pET-26b
human FADD_DED reverse	CGTACTCGAGCGCCTCGAAGTCGTCGACGC	pET-26b
human FADD reverse	CGTACTCGAGAGACGCTTCGGAGGTAGATGC	pET-26b
Caspase-8 forward	GATCCATATGGACTTCAGCAGAAATCTTTATGATATTG	pET-26b
Caspase-8_tDED reverse	CTAGCTCGAGTTATCTCTCTTTGCTGAATTCTTCATAGT CGTTG	pET-26b
Caspase-8_FL reverse	CGTAGCGGCCGCTCAATCAGAAGGGAAGACAAGTTTTT TTC	pET-26b
cFLIP forward	GATCCATATGTCTGCTGAAGTCATCCATCAGGTTG	pET-26b
cFLIP_tDED reverse	CGTACTCGAGCTAACTTGTCCCTGCTCCTTGAACAGACT G	pET-26b
cFLIP_tDED reverse2	CGTACTCGAGACTTGTCCCTGCTCCTTGAACAGACTG	pET-26b

Mutagenesis primers		
FADD_H9G forward	GGCTCGGTGTCGTCCAGCCTGTCGAGC	pET-26b
FADD_H9G reverse	CAGCAGCACCAGGAACGGGTCCAT	pET-26b
FADD_F25Y forward	CGAGCTGACCGAGCTCAAGTACCTATGCCTCGGGCGCG TGG	pET-26b
FADD_F25Y reverse	CCACGCGCCCGAGGCATAGGTACTTGAGCTCGGTCAGC TCG	pET-26b
FADD_F25G forward	GGCCTATGCCTCGGGCGCGTGGG	pET-26b
FADD_F25G reverse	CTTGAGCTCGGTCAGCTCGCTGCTCG	pET-26b
FADD_D74K forward	AAGCTGCTGCGGCGCGTCGACG	pET-26b
FADD_D74K reverse	GTGGCGCCGCAGGGAGGCG	pET-26b
Caspase-8_F122A forward	CAGAATTGAGGTCTTTTAAGGCTCTTTTGCAAGAGGAA ATCTCC	pET-26b
Caspase-8_F122A reverse	GGAGATTTCCTCTTGCAAAAGAGCCTTAAAAGACCTCA ATTCTG	pET-26b
Caspase-8_F122G\L123G forward	GAATTGAGGTCTTTTAAGGGCGGTTTGCAAGAGGAAAT CTCC	pET-26b
Caspase-8_F122G\L123G reverse	GGAGATTTCCTCTTGCAAACCGCCCTTAAAAGACCTCA ATTC	pET-26b
Caspase-8_C360A F	GCTCAGGGGGATAACTACCAGAAAGGTATACCTG	pET-26b
Caspase-8_C360A R	AGCCTGAATAAAAAACACTTTGGGTTTTCCAGC	pET-26b
Caspase-8_D374A F	GCCTCAGAGGAGCAACCCTATTTAGAAATGGATTTATC	pET-26b
Caspase-8_D374A R	AGTCTCAACAGGTATACCTTTCTGGTAGTTATCCCC	pET-26b
Caspase-8_D384A_R	GCATTATCATCACCTCAAACGAGATATATCCCGG	pET-26b
Caspase-8_D384A_F	CATTTCTAAATAGGGTTGCTCCTCTGAGGCAGTC	pET-26b

cFLIP_H7G forward	CATATGTCTGCTGAAGTCATCGGTCAGGTTGAAGAAGC ACTTG	pET-26b
cFLIP_H7G reverse	CAAGTGCTTCTTCAACCTGACCGATGACTTCAGCAGAC ATATG	pET-26b
cFLIP_F114G forward	GGCCTCATGAAGGATTACATGGGCCG	pET-26b
cFLIP_F114G reverse	AATTAATGAGGACACATCAGATTTATCCAAATCCTCAC	pET-26b, pAS4.1w.Pbsd-aOn
hFAD_R34A_inF_F	GGG CAA GGC CAA GCT GGA GCG CGT GCA G	pET-26b, pAS4.1w.Pbsd-aOn
hFAD_R34A_inF_R	AGC TTG GCC TTG CCC ACG CGC CCG AG	pET-26b, pAS4.1w.Pbsd-aOn
hFAD_E22A_inF_F	GCT GAC CGC CCT CAA GTT CCT ATG CCT CGG GC	pET-26b, pAS4.1w.Pbsd-aOn
hFAD_E22A_inF_R	TTG AGG GCG GTC AGC TCG CTG CTC GAC	pET-26b, pAS4.1w.Pbsd-aOn
hFAD_F25R_inF_F	GCT CAA GCG CCT ATG CCT CGG GCG CGT GG	pET-26b, pAS4.1w.Pbsd-aOn
hFAD_F25R_inF_R	CAT AGG CGC TTG AGC TCG GTC AGC TCG C	pET-26b, pAS4.1w.Pbsd-aOn
hFAD_D74A_inF_F	GCG CCA CGC CCT GCT GCG GCG CGT CGA CG	pET-26b, pAS4.1w.Pbsd-aOn
hFAD_D74A_inF_R	AGC AGG GCG TGG CGC CGC AGG GAG GC	pET-26b, pAS4.1w.Pbsd-aOn
hFAD_R34A_inF_F	GGG CAA GGC CAA GCT GGA GCG CGT GCA G	pET-26b, pAS4.1w.Pbsd-aOn
hFAD_R34A_inF_R	AGC TTG GCC TTG CCC ACG CGC CCG AG	pET-26b, pAS4.1w.Pbsd-aOn
hFAD_K33E_inF_F	CGT GGG CGA ACG CAA GCT GGA GCG CGT G	pET-26b, pAS4.1w.Pbsd-aOn
hFAD_K33E_inF_R	TTG CGT TCG CCC ACG CGC CCG AGG CAT AG	pET-26b, pAS4.1w.Pbsd-aOn
hFAD_E37A_inF_F	CAA GCT GGC CCG CGT GCA GAG CGG CCT AG	pET-26b, pAS4.1w.Pbsd-aOn
hFAD_E37A_inF_R	ACG CGG GCC AGC TTG CGC TTG CCC AC	pET-26b, pAS4.1w.Pbsd-aOn
hFAD_E51R_inF_F	GCT GCT GCG CCA GAA CGA CCT GGA GCC C	pET-26b, pAS4.1w.Pbsd-aOn
hFAD_E51R_inF_R	TTC TGG CGC AGC AGC ATG GAG AAG AGG	pET-26b, pAS4.1w.Pbsd-aOn
hFAD_Q40A_inF_F	GCG CGT GGC CAG CGG CCT AGA CCT CTT CTC C	pET-26b, pAS4.1w.Pbsd-aOn
hFAD_Q40A_inF_R	CCG CTG GCC ACG CGC TCC AGC TTG CG	pET-26b, pAS4.1w.Pbsd-aOn
mFAD_E22A_inF_F	TCT GAT GGC CCT CAA GTT CTT GTG CCG CGA	pAS4.1w.Pbsd-aOn
mFAD_E22A_inF_R	TTG AGG GCC ATC AGA TCG TTG CCC GAC A	pAS4.1w.Pbsd-aOn
mFAD_F25R_inF_F	GCT CAA GCG CTT GTG CCG CGA GCG CGT GAG C	pAS4.1w.Pbsd-aOn
mFAD_F25R_inF_R	CAC AAG CGC TTG AGC TCC ATC AGA TCG TTG CC	pAS4.1w.Pbsd-aOn
mFAD_D74A_inF_F	CCG ACA CGC CCT ACT GCA GCG CCT GGA CG	pAS4.1w.Pbsd-aOn
mFAD_D74A_inF_R	AGT AGG GCG TGT CGG CGC AGC GAG GC	pAS4.1w.Pbsd-aOn
mFAD_R34A_inF_F	GAG CAA AGC CAA GCT GGA GCG CGT GCA GAG TGG	pAS4.1w.Pbsd-aOn
mFAD_R34A_inF_R	AGC TTG GCT TTG CTC ACG CGC TCG CG	pAS4.1w.Pbsd-aOn
mFAD_K33E_inF_F	CGT GAG CGA ACG AAA GCT GGA GCG CGT G	pAS4.1w.Pbsd-aOn
mFAD_K33E_inF_R	TTT CGT TCG CTC ACG CGC TCG CGG CAC	pAS4.1w.Pbsd-aOn
mFAD_E37A_inF_F	AAA GCT GGC CCG CGT GCA GAG TGG CCT G	pAS4.1w.Pbsd-aOn
mFAD_E37A_inF_R	ACG CGG GCC AGC TTT CGT TTG CTC ACG CG	pAS4.1w.Pbsd-aOn
mFAD_E51R_inF_F	GCT GCT GCG CCA GAA CGA CCT GGA GCG C	pAS4.1w.Pbsd-aOn
mFAD_E51R_inF_R	TTC TGG CGC AGC AGC ACC GTG AAC AGG	pAS4.1w.Pbsd-aOn
mFAD_Q40A_inF_F	GCG CGT GGC CAG TGG CCT GGA CCT GTT CAC	pAS4.1w.Pbsd-aOn
mFAD_Q40A_inF_R	CCA CTG GCC ACG CGC TCC AGC TTT CG	pAS4.1w.Pbsd-aOn

Supplementary References:

1. Fu TM*, et al.* Cryo-EM Structure of Caspase-8 Tandem DED Filament Reveals Assembly and Regulation Mechanisms of the Death-Inducing Signaling Complex. *Mol Cell* **64**, 236-250 (2016).

2. Fox JL*, et al.* Cryo-EM structural analysis of FADD:Caspase-8 complexes defines the catalytic dimer architecture for co-ordinated control of cell fate. *Nat Commun* **12**, 819 (2021).

3. Hopkins JB. BioXTAS RAW 2: new developments for a free open-source program for small-angle scattering data reduction and analysis. *J Appl Crystallogr* **57**, 194-208 (2024).
