# Supplementary material for: Deciphering DED assembly mechanisms in FADD-procaspase-8-cFLIP complexes regulating apoptosis
Source: Nat Commun. 2024 May 6;15:3791. doi: 10.1038/s41467-024-47990-2 (PMC11074299; doi:10.1038/s41467-024-47990-2)
Supplement: Supplementary file 3 — Description of Additional Supplementary Files [file 41467_2024_47990_MOESM3_ESM.pdf]

Description of Additional Supplementary Files

File Name: Supplementary Movie 1
Description: The side and top/bottom views of the crystal structure of the singleFADD complex. Shows the side, top, and bottom views of the crystal structure of the single-FADD complex, in which FADD^{DED} molecule is colored in red, while cFLIPt^{DED} molecules are colored in pink. Casp8t^{DED} molecule are colored as those in Fig.2a.

File Name: Supplementary Movie 2
Description: The cryo-EM volume or structure of the triple-FADD complex with the atomic coordinates. Shows the side, top, and bottom views of cryo-EM volume or structure of the triple-FADD complex. This movie also shows that the corresponding atomic coordinates for FADD, cFLIP, and Casp-8 DED could fit the cryo-EM structure. The atomic coordinates for FADD, cFLIP, and Casp8 DED are colored as those in Fig.2b. For comparison, please see Supplementary Movie 8.

File Name: Supplementary Movie 3
Description: FADD DED binds a Casp-8 tDED helical pentamer in the single-FADD complex. Shows that, after removing cFLIPt^{DED} and FADD^{DED} molecule,shown in pink and red, respectively, of the crystal structure of the single-FADD complex, remaining Casp-8t^{DED} molecules assemble a helical pentamer via the type III-II-III CSS. Therefore, the Casp-8t^{DED} pentamer is stabilized by a FADD^{DED} molecule (red), resulting in a FADD-Casp-8 intermediate complex. The surface of Casp-8 tDED and some type I, II, III surfaces were colored as those in Fig. 3.

File Name: Supplementary Movie 4
Description: The single-FADD-Casp-8 intermediate complex. Similar to Supplementary Movie 3, but shows only the single-FADD-Casp-8 intermediate complex derived from our crystal structure.

File Name: Supplementary Movie 5
Description: The FADD-Casp-8 intermediate complex binds four cFLIPt^{DED} molecules. Shows the FADD-Casp-8 intermediate complex assembled in Supplementary Movie 3 binds four cFLIPt^{DED} molecules, resulting in the crystal structure of the single-FADD complex.

File Name: Supplementary Movie 6
Description: cFLIP targets the single-FADD-Casp-8 intermediate complex. Similar to Supplementary Movie 5, but shows that four cFLIPt^{DED} molecules, shown in pink, are sequentially recruited to the complex by targeting the CBS appearing on the FADD-Casp-8 intermediate complex and the CBS appearing later on subsequent FADD-Casp-8-cFLIP complex intermediates or products.

File Name: Supplementary Movie 7
Description: The single-FADD-Casp-8 intermediate complex binds Casp-8 when cFLIP is depleted. Shows that the CBS of the single-FADD-Casp-8 intermediate complex could recruit Casp-8t^{DED}. Three Casp-8t^{DED} molecules (blue) are sequentially recruited in order to complete an asymmetric unit, also the second Casp-8t^{DED} layer of a potentially growing Casp-8t^{DED} filament. Notably, the resultant Casp-8t^{DED} double layer would assemble a CBS to recruit the fourth Casp-8t^{DED} molecule, with green edge glow in the movie. By repeating this process, more Casp-8t^{DED} molecules could join this complex to extend the Casp-8t^{DED} filament.

File Name: Supplementary Movie 8
Description: Comparison of the cryo-EM volume (EMD-11941) with our singleFADD model. The single-FADD model is colored as that in Fig. 2a. For comparison, please see Supplementary Movie 2. The movie illustrates the challenges encountered in fitting and determining the orientation of the atomic coordinates of an oligomeric DED/tDED complex within the cryo-EM volume of EMD-11941 [https://www.ebi.ac.uk/pdbe/entry/emdb/EMD-11941], using our singleFADD model as an example. The single-FADD model is colored as that in Fig. 2a. For comparison, please refer to Supplementary Movie 2.
