# Supplementary material for: Deciphering DED assembly mechanisms in FADD-procaspase-8-cFLIP complexes regulating apoptosis
Source: Nat Commun. 2024 May 6;15:3791. doi: 10.1038/s41467-024-47990-2 (PMC11074299; doi:10.1038/s41467-024-47990-2)
Supplement: Supplementary file 12 — Reporting Summary [file 41467_2024_47990_MOESM12_ESM.pdf]

nature portfolio

Corresponding author(s): Su-Chang Lin, Yu-Chih Lo, Li-Chung Hsu

Last updated by author(s): Apr 9, 2024

Reporting Summary

Nature Portfolio wishes to improve the reproducibility of the work that we publish. This form provides structure for consistency and transparency in reporting. For further information on Nature Portfolio policies, see our Editorial Policies and the Editorial Policy Checklist.

Statistics

For all statistical analyses, confirm that the following items are present in the figure legend, table legend, main text, or Methods section.

n/a	Confirmed	
☐	☒	The exact sample size (n) for each experimental group/condition, given as a discrete number and unit of measurement
☐	☒	A statement on whether measurements were taken from distinct samples or whether the same sample was measured repeatedly
☒	☐	The statistical test(s) used AND whether they are one- or two-sided<br>*Only common tests should be described solely by name; describe more complex techniques in the Methods section.*
☒	☐	A description of all covariates tested
☒	☐	A description of any assumptions or corrections, such as tests of normality and adjustment for multiple comparisons
☐	☒	A full description of the statistical parameters including central tendency (e.g. means) or other basic estimates (e.g. regression coefficient) AND variation (e.g. standard deviation) or associated estimates of uncertainty (e.g. confidence intervals)
☒	☐	For null hypothesis testing, the test statistic (e.g. F, t, r) with confidence intervals, effect sizes, degrees of freedom and P value noted<br>*Give P values as exact values whenever suitable.*
☒	☐	For Bayesian analysis, information on the choice of priors and Markov chain Monte Carlo settings
☒	☐	For hierarchical and complex designs, identification of the appropriate level for tests and full reporting of outcomes
☒	☐	Estimates of effect sizes (e.g. Cohen's d, Pearson's r), indicating how they were calculated

Our web collection on statistics for biologists contains articles on many of the points above.

Software and code

Policy information about availability of computer code

Data collection	EPU 2.2.0.65REL was used for cryo-EM data collection. X-ray diffraction data was collected by the program Blu-Ice 5.0. SAXS data was collected using the program Albula (Dectris, Baden-Dättwil, Switzerland).
Data analysis	cryo-EM data was processed by using CryoSparc V2.8.0 to V2.11.0, X-ray data was processed by using HKL2000, and the structure was solved and refined by using Phenix 1.14-3260. The structures were adjusted by using Coot 0.8.9.1 EL or WinCoot 0.8.2. The structures were analyzed or portrayed by using the qtPISA program in the CCP4i2 program suit 7.0.060, MUSTANG 3.2.3, Chimera 1.13.1, ChimeraX 0.91, PyMOL 1.8.2.1, HELANAL-Plus (https://dna.mbu.iisc.ac.in/resources.htm), and MolProbity (http://kinemage.biochem.duke.edu). The bands on the SDS-PAGE were analyzed by ImageJ 1.50i. The SAXS data was processed and analyzed by RAW 2.2.2 and the ATSAS program suite 2.7, 2.8.1, and 3.2.1. MALS aata analysis was carried out using the program ASTRA ver 6.0.5.3. Bar charts were made by GraphPad Prism 9.4.1

For manuscripts utilizing custom algorithms or software that are central to the research but not yet described in published literature, software must be made available to editors and reviewers. We strongly encourage code deposition in a community repository (e.g. GitHub). See the Nature Portfolio guidelines for submitting code & software for further information.

Data

Policy information about availability of data

All manuscripts must include a data availability statement. This statement should provide the following information, where applicable:

- Accession codes, unique identifiers, or web links for publicly available datasets
- A description of any restrictions on data availability
- For clinical datasets or third party data, please ensure that the statement adheres to our policy

The atomic coordinates and reflection files for the crystal structures generated in this study have been deposited in the Worldwide Protein Data Bank (wwPDB) under accession codes 8YD7 [http://doi.org/10.2210/pdb8YD7/pdb] (The SeMet derivative of the single-FADD-Casp-8-cFLIP DED complex) and 8YD8 [http://doi.org/10.2210/pdb8YD8/pdb] (Native single-FADD-Casp-8-cFLIP DED complex).

The cryo-EM structures generated in this study have been deposited in the Electron Microscopy Data Bank (EMDB) under accession codes EMD-39126 [https://www.ebi.ac.uk/pdbe/entry/emdb/EMD-39126] (The triple-FADD-Casp-8-cFLIP DED complex B) and EMD-39127 [https://www.ebi.ac.uk/pdbe/entry/emdb/EMD-39127] (The triple-FADD-Casp-8-cFLIP DED complex A).

The atomic coordinates for the cryo-EM structure generated in this study have been deposited in the wwPDB under accession code 8YBX [http://doi.org/10.2210/pdb8YBX/pdb] (The triple-FADD-Casp-8-cFLIP DED complex B).

The cryo-EM structure used in this study are available in the EMDB under accession codes EMD-11939 [https://www.ebi.ac.uk/pdbe/entry/emdb/EMD-11939] (Central region of Caspase-8:FADD complex), and EMD-11941 [https://www.ebi.ac.uk/pdbe/entry/emdb/EMD-11941] (The ternary complex of full-length Caspase-8 with FADD and FLIPs).

The atomic coordinates for the cryo-EM structure used in this study are available in the wwPDB under accession codes 5L08 [http://doi.org/10.2210/pdb5L08/pdb] (Casp-8 tDED filaments).

All other data generated in this study are provided in the Supplementary Information or a Source Data file. Alternatively, any data that support this study are also available from the corresponding authors upon request.

Research involving human participants, their data, or biological material

Policy information about studies with human participants or human data. See also policy information about sex, gender (identity/presentation), and sexual orientation and race, ethnicity and racism.

Reporting on sex and gender	not applicable (This data didn't involve human participants or human data.)
Reporting on race, ethnicity, or other socially relevant groupings	not applicable (This data didn't involve human participants or human data.)
Population characteristics	not applicable (This data didn't involve human participants or human data.)
Recruitment	not applicable (This data didn't involve human participants or human data.)
Ethics oversight	not applicable (This data didn't involve human participants or human data.)

Note that full information on the approval of the study protocol must also be provided in the manuscript.

Field-specific reporting

Please select the one below that is the best fit for your research. If you are not sure, read the appropriate sections before making your selection.

[x] Life sciences [ ] Behavioural & social sciences [ ] Ecological, evolutionary & environmental sciences

For a reference copy of the document with all sections, see nature.com/documents/nr-reporting-summary-flat.pdf

Life sciences study design

All studies must disclose on these points even when the disclosure is negative.

Sample size	To obtain atomic coordinates by cryo-EM studies, the size of sample would depend on: 1, whether the image quality is good. 2, whether the particle density and the number of the particles are high enough for data analysis. 3, whether the particles are homogeneous. 4, whether the angular distribution of the particles is good. 5. whether the resolution of the reconstructed 3D volume is sufficient to atomic model building. 6, whether quality of the atomic coordinates is good.<br><br>As shown in Supplementary Fig. 3 and Supplementary Table 3, 3,010 movies were collected to produce ~356,000 particles of Complex B. ~28,000 particles were used to reconstruct the final 3D volume of Complex B to a resolution of 3.7 angstrom, based on the FSC threshold of 0.143. The production of the atomic coordinates at a resolution of 3.7 angstrom indicates that the sample sizes are sufficient, as supported by the validation reports.

To obtain atomic coordinates by X-ray crystallography, whether the sample size is sufficient depends on: 1, whether the Signal-to-Noise ratio of the reflections is good enough. 2, whether the resolution is sufficient to atomic model building. 3, whether the quality of the reflection data is good. 4, whether the completeness of the data is good. 5, whether the data redundancy is enough. 6, whether the refinement statistics are good.

In this study, 53,898 reflections, with a redundancy of 7.8, completeness of 99.7, and average I/sigma of 21.65, were used to obtain the crystal structure of the single-FADD complex at a resolution of 3.1 angstrom. The X-ray Data collection, phasing and refinement statistics in Supplementary Table 1 indicate that the sample sizes are sufficient, which is supported by the validation report.

Biochemical experiments, such as cloning, protein expression and purification, and crystallization, were conducted by following repeatedly standard practices in order to reach desired quality of products, as demonstrated by DNA sequencing, SDS-PAGE, and X-ray diffraction, respectively. Therefore, no sample-size determination was performed. The sample sizes for other biochemical experiments were chosen to be n=2 or higher for consistent results.

Data exclusions	For cryo-EM data, according to standard practices, movies with image drift, ice contamination, or poor CTF estimation were excluded. In addition, bad particles with no secondary structural features in 2D classification were excluded. For X-ray data, according to standard practices, poor diffraction images and bad reflections would be excluded.
Replication	Cryo-EM or EM experiments were performed independently at least three times with similar results or with similar resultant structures of different resolutions. The reproducibility could also be evaluated and verified by statistical analyses (Supplementary Table 3) and the validation report. In addition, the reproducibility could be verified by the fact that GraFix-stabilized and non-crosslinked versions of the cryo-EM samples led to the same 3D structures of Complexes B and A, respectively, with different resolutions. Furthermore, the 3D volume of Complex B, for example, was reconstructed by 28,000 particles to a resolution of 3.7 angstrom, indicating that ~28,000 particles consistently have the same structure at a resolution of 3.7 angstrom.<br><br>X-ray diffraction experiments were data was performed independently at least three times with similar crystal parameters or with similar resultant X-ray structures of different resolutions. The reproducibility could also be evaluated and verified by statistical analyses (Supplementary Table 1) and the validation report. In addition, the reproducibility could be verified by the same structures produced by the native complex and its SeMet derivative. Furthermore, since the single crystal used in each X-ray diffraction experiment contains at least a billion protein molecules, the generation of the structure of the single-FADD complex a resolution of 3.1 angstrom indicates the a least a billion protein molecules in the crystal have the same structure at a resolution of 3.1 angstrom.<br><br>Other biochemical experiments were repeated at least twice with similar results.
Randomization	Particles in the refinement step of cryo-EM data processing were randomly split into two halves by the software automatically and cross correlation coefficients between the two half sets are calculated. For the refinement of X-ray structure, Phenix would randomly pick 5% of reflections to calculate R-free. No randomization was involved in other biochemical experiments if the samples are from the same strain or the same flask.
Blinding	iMEF cells and E. coli for experiments are derived from the same batch. Investigators were blinded to group allocation. Blinding is not applicable to other experiments, such as cryo-EM and X-ray diffraction, because the measurements are objective and easily measurable. Therefore, the parameters for the experiments in this study did not require subjective assessments of the treatment.

Reporting for specific materials, systems and methods

We require information from authors about some types of materials, experimental systems and methods used in many studies. Here, indicate whether each material, system or method listed is relevant to your study. If you are not sure if a list item applies to your research, read the appropriate section before selecting a response.

Materials & experimental systems

n/a	Involved in the study	
☐	☒	Antibodies
☐	☒	Eukaryotic cell lines
☒	☐	Palaeontology and archaeology
☒	☐	Animals and other organisms
☒	☐	Clinical data
☒	☐	Dual use research of concern
☒	☐	Plants

Methods

n/a	Involved in the study	
☒	☐	ChIP-seq
☒	☐	Flow cytometry
☒	☐	MRI-based neuroimaging

Antibodies

Antibodies used	Cell-based experiments: phospho-IKKalpha/beta(Ser176/180) (Cell Signaling, #2697L, 1:1000); IKKalpha/IKKbeta (Santa Cruz, #sc-7607,1:1000); GAPDH (GeneTex, #GTX627408, 1:5000); cFLIP (dave-2) (Adipogen, #AG-20B-0005, 1:1000); Caspase 8 (D35G2) (Cell Signaling, #4790, 1:1000); Cleaved Caspase-3 (Asp175) (5A1E) (Cell Signaling, #9664, 1:1000); PARP (Cell Signaling, #9532,1:1000); phospho-MLKL (S345) (Abcam, #ab196436, 1:2000); MLKL (clone 3H1) (Millipore, #MABC604, 1:1000); phospho-RIP3 (Thr231/Ser232) (Cell Signaling, #57220, 1:1000); RIP3 (Cell Signaling, #95702, 1:1000); RIP1 (BD, #610459, 1:2000); Phospho-RIP (Ser166) (Cell Signaling, #31122, 1:1000); phospho-p38 (Thr180/Tyr182) (Cell Signaling, #9211,1:1000); p38 MAPK (Cell Signaling,

	#9212, 1:1000); phospho-JNK/SAPK (Thr183/Tyr185) (Cell Signaling, #9251, 1:1000); JNK/SAPK (Cell Signaling, #9252, 1:1000); FADD (Abcam, #Ab124812, 1:1000); FADD (human specific) (Cell Signaling, #2782, 1:1000).<br>Cell lysate-based experiments: FLIP (D5J1E) (Cell Signaling, #56343, 1:2000); Caspase 8(D35G2) (Cell Signaling, #4790, 1:4000); Caspase-3 Antibody (Cell Signaling, #9662, 1:4000); Cleaved Caspase-3 (Asp175) (5A1E) (Cell Signaling, #9664, 1:2000); Human FADD (Cell Signaling, #2782, 1:2000); PARP (46D11) (Cell Signaling, #9532,1:2000); RIP (Cell Signaling, #4926, 1:2000); Phospho-RIP (Ser166) (D1L3S) (Cell Signaling, #65746, 1:2000); GAPDH (14C10) (HRP Conjugate) (Cell Signaling, #3683, 1:4000); Goat Anti-Rabbit IgG H&L (HRP) (Abcam, #ab6721, 1:10000)
Validation	Primary antibodies were validated by the manufacturers as shown on the corresponding websites. All the antibody are available from the commercial sources listed above. All the RRID are listed below:<br>phospho-IKKalpha/beta(Ser176/180) (Cell Signaling, #2697L)RRID:AB_2079382;<br>IKKalpha/IKKbeta (Santa Cruz, #sc-7607)RRID:AB_675667;<br>GAPDH (GeneTex, #GTX627408) RRID:AB_11174761;<br>cFLIP (dave-2) (Adipogen, #AG-20B-0005)RRID:AB_2490185;<br>Caspase 8 (D35G2) (Cell Signaling, #4790)RRID:AB_10545768;<br>Cleaved Caspase-3 (Asp175) (5A1E) (Cell Signaling, #9664)RRID:AB_2070042;<br>PARP (Cell Signaling, #9532)RRID:AB_659884;<br>phospho-MLKL (S345) (Abcam, #ab196436)RRID:AB_2687465;<br>MLKL (clone 3H1) (Millipore, #MABC604)RRID:AB_2820284;<br>phospho-RIP3 (Thr231/Ser232) (Cell Signaling, #57220)RRID:AB_2799526;<br>RIP3 (Cell Signaling, #95702)RRID:AB_2721823;<br>RIP1 (BD, #610459)RRID:AB_397832;<br>Phospho-RIP (Ser166) (Cell Signaling, #31122)RRID:AB_2799000;<br>phospho-p38 (Thr180/Tyr182) (Cell Signaling, #9211)RRID:AB_331641;<br>p38 MAPK (Cell Signaling, #9212)RRID:AB_330713;<br>phospho-JNK/SAPK (Thr183/Tyr185) (Cell Signaling, #9251)RRID:AB_331659;<br>JNK/SAPK (Cell Signaling, #9252)RRID:AB_2250373<br>FADD (Abcam, #Ab124812)RRID:AB_10976310<br>FADD (human specific) (Cell Signaling, #2782)RRID:AB_2100484;<br>FLIP (D5J1E) (Cell Signaling, #56343)RRID:AB_2799508;<br>Caspase-3 Antibody (Cell Signaling, #9662)RRID:AB_331439;<br>RIP (Cell Signaling, #4926)RRID:AB_2224503;<br>Phospho-RIP (Ser166) (D1L3S) (Cell Signaling, #65746)RRID:AB_2799693;<br>GAPDH (14C10) (HRP Conjugate) (Cell Signaling, #3683)RRID:AB_1642205;<br>Goat Anti-Rabbit IgG H&L (HRP) (Abcam, #ab6721)RRID:AB_955447

Eukaryotic cell lines

Policy information about cell lines and Sex and Gender in Research

Cell line source(s)	HEK293T cells were obtained from ATCC (CRL-3216)RRID:CVCL_0063; FADD-KO HAP1 cells (RRID:CVCL_SM74) were purchased from Horizon Discovery Group plc; Primary Mouse Embryonic Fibroblasts (MEFs) were generated by L.C.H.'s lab according to the previous protocol (Current Protocols in Molecular Biology (2005) 28.1.1-28.1.8) and used in J Exp Med (2023) 220 (8): e20220727. https://doi.org/10.1084/jem.20220727.
Authentication	These cell lines were not authenticated by ourself in this study.<br>Cell lines directly from the commercial sources should be authenticated by the suppliers, including<br>HEK293T cells (CRL-3216)RRID:CVCL_0063 and FADD-KO HAP1 cells, RRID:CVCL_SM74).
Mycoplasma contamination	All cell lines tested negative for mycoplasma contamination. Mycoplasma contamination in cell cultures was routinely detected by PCR using the primes, forward primer: 5'-GGGAGCAAACACGATAGATACCCT-3' and reverse primer: 5'-TGCACCATCTGTCACTCTGTTAACCTC-3', as suggested previously (Timenetsky, J., Santos, L. M., Buzinhani, M. & Mettifogo, E. Braz. J. Med. Biol. Res.2006; 39, 907–914).
Commonly misidentified lines<br>(See ICLAC register)	No commonly misidentified cell lines were used in this study.

Plants

Seed stocks	n/a
Novel plant genotypes	n/a
Authentication	n/a
